# Crop rotation complexity affects soil properties shaping antibiotic resistance gene types and resistance mechanisms

**DOI:** 10.3389/fmicb.2025.1603518

**Published:** 2025-06-25

**Authors:** Rong Hu, Yang Liu, Mengmeng Wen, Nafang Zhou, Jun Wang

**Affiliations:** ^1^Shaanxi Key Laboratory of Earth Surface System and Environmental Carrying Capacity, College of Urban and Environmental Science, Northwest University, Xi’an, China; ^2^Shaanxi Key Laboratory for Carbon Neutral Technology, Northwest University, Xi’an, China

**Keywords:** antibiotic resistance genes, crop rotation complexity, crop rotation regimes, Loess Plateau, resistance mechanisms, winter wheat field

## Abstract

Crop rotation enhances agricultural productivity and soil fertility but may also contribute to the accumulation of antibiotic resistance genes (ARGs). However, the changes in soil ARGs and their associated resistance mechanisms under different crop rotation regimes are not well understood. In this study, we employed metagenomics to comprehensively investigate soil ARGs under different crop rotation regimes and complexity. Our findings revealed that soil properties varied significantly with crop rotation regime and complexity. Specifically, soil pH and the total carbon/nitrogen ratio (C/N) were the highest in bare land (BL) and gradually decreased in the order non-rotation, simple rotation, and complex rotation systems. The composition of soil ARGs exhibited significant differentiation by crop rotation complexity. Furthermore, differential gene analysis identified four specific types of ARGs—glycopeptide, multidrug, fluoroquinolone, and macrolide-lincosamide-streptogramin B (MLSB)—and two resistance mechanisms—cellular protection and efflux pump. Notably, soil microbial biomass carbon, soil microbial biomass nitrogen, and soil organic carbon are significantly correlated with ARGs in complex crop rotation systems, whereas soil pH and C/N ratio show significant associations in BL. The C/N ratio was identified as the most relevant determinant for glycopeptide, multidrug, fluoroquinolone, and MLSB resistance genes. Overall, these findings elucidate key factors associated with ARGs under long-term crop rotation, thereby providing valuable insights into the influence of crop rotation regimes on soil ARGs and enhancing soil fertility by improving soil properties.

## 1 Introduction

ARGs have become ubiquitous in various environmental compartments including airborne particulate matter, wastewater treatment sludge (Xie et al., 2016), and soil, posing significant threats to public health through their widespread dissemination (Wang et al., 2023). In recent years, a diverse array of ARGs has been detected in agricultural soils worldwide (Ondon et al., 2021; Shen et al., 2023). Notably, as a critical reservoir for ARGs (Khalid et al., 2023), soil acquires resistance genes through dual pathways (Su et al., 2014; Wang et al., 2023): intrinsic microbial carriage (Han et al., 2023) and exogenous inputs from agricultural activities (Wang et al., 2018, 2023; Gao et al., 2023; Han et al., 2023). Fertilization and irrigation have been demonstrated to influence the abundance and dissemination of ARGs by altering microbial communities, nutrient dynamics, and cross-ecosystem transmission pathways (Xie et al., 2016; Wang et al., 2023). However, the long-term effects of crop rotation—a crucial anthropogenic intervention—on ARGs remain insufficiently understood.

Research has demonstrated that crop rotation regimes profoundly influence the soil ARGs (Zhang et al., 2021). The abundance of sulfonamide genes was found to be higher in maize fields compared to wheat fields under wheat–maize rotation systems (Xu et al., 2021). In monoculture cropping systems, the abundance of resistance genes also varied depending on the cropping system (Huang et al., 2021). Crop diversification typically enhances microbial abundance (Liu et al., 2024), providing potential microbial carriers for soil ARGs. Alterations in the composition and function of microbial communities—a crucial component of soil ecosystems—determine the distribution, dissemination, and evolution of soil ARGs (Zhang et al., 2023). Crop rotation systems alter the diversity and distribution of soil ARGs by cultivating a variety of plant species (Venter et al., 2016; Huang et al., 2021). Most existing studies focus on short-term effects in simple rotation systems. However, the cumulative effects in complex (multi-species, long-term) systems remain unclear, limiting our ability to predict the ecological risks of ARGs under realistic agricultural practices. Although complex and diverse crop rotation systems substantially enhance agricultural productivity and soil fertility (Bowles et al., 2020, 2022), they also pose the potential risk of accelerating the accumulation of ARGs. A comprehensive investigation into the impacts of long-term, complex rotation systems on ARGs is crucial for optimizing agricultural benefits while mitigating the associated ecological and public health risks.

Environmental factors are closely associated with ARGs. In surface soils with long-term pig manure application, heavy metals were found to be significantly positively correlated with the abundance of ARGs (Guo et al., 2018). Microbial carbon and nitrogen (MBC and MBN) have been identified as important predictors of changes in ARG patterns (Li et al., 2022). In greenhouse soils, total nitrogen and organic matter emerge as the two major soil properties affecting the abundance of ARGs (Wang et al., 2020). Moreover, the C/N ratio has been proven to regulate ARG removal during the composting process (Zhu et al., 2021). These studies highlight the influence of environmental factors on ARGs in different soils. However, the relationship between environmental factors and ARGs under crop rotation systems requires further investigation. Notably, most previous studies have relied on quantitative PCR and amplicon sequencing to determine the abundance and distribution of soil ARGs (Li et al., 2020). In contrast, metagenomic sequencing provides a more comprehensive approach by capturing the genomic diversity of environmental microbial communities, enabling the identification of novel ARGs and tracing their transmission pathways (Marić and Šikić, 2019). However, studies combining metagenomics with long-term field experiments to investigate ARGs in arid agricultural ecosystems have not yet been reported.

The Loess Plateau of China is a typical arid and semi-arid region. Its unique soil and water conditions may create distinctive distribution patterns of ARGs. Crop rotation and diversification are important agricultural practices in this region. However, existing studies in this region have primarily focused on the effects of crop rotation on soil fertility (Liu Y. et al., 2023), with limited attention to the impact of crop rotation on ARGs as emerging contaminants. Therefore, this study aims to: (1) evaluate the effects of different crop rotation systems on soil properties and microbial biomass; (2) investigate the abundance, diversity, and resistance mechanisms of ARGs under long-term complex crop rotation regimes using metagenomic analysis; and (3) identify the factors associated with ARGs under crop rotation regimes and complexity.

## 2 Materials and methods

### 2.1 Experimental site description and soil sampling

The crop rotation experiment was initiated in September 1984 in a wheat field, with the site located at the Loess Plateau Agricultural Ecology Experimental Station (35°12′N, 107°44′E) of the Chinese Academy of Sciences (Changwu County, Shaanxi Province, China) (Cai and Hao, 2015; Fu et al., 2019; Liu Y. et al., 2023). This site is situated in the East Asian continental monsoon climate zone, with an average annual temperature of 9.1°C and 580 mm of precipitation. The soil is classified as Heilutu silt loam (Calcarid Regosol according to the Food and Agriculture Organization of the United Nations classification system), with 45 g kg^−1^ sand, 656 g kg^−1^ silt and 309 g kg^−1^ clay at depth of 0–20 cm at the beginning of the experiment (Fu et al., 2019).

In this study, we set up five different crop rotation regimes: bare land (BL), winter wheat monoculture (W), pea–winter wheat–winter wheat–millet (PWWM), corn–winter wheat–winter wheat–millet (CWWM), and alfalfa (4 yr) –potato (1 yr) –winter wheat (3 yr) (A4PoW3). We calculated the rotation complexity index (RCI) for these regimes, which was defined as the square root of the number of cash and cover crop species (crop species richness) in a rotation multiplied by the length of the rotation (crop cover duration). The five rotation regimes were classified into four crop rotation complexity levels based on RCI: bare land (BL), non-rotation (W, RCI = 1), simple rotation (PWWM and CWWM, RCI = 3), and complex rotation (A4PoW3, RCI = 5) (Bowles et al., 2020, 2022). The experiment was arranged in a completely randomized block design with three replications over five crop rotations with a rotation length of 1–10 years (Fu et al., 2019; Liu Y. et al., 2023).

Samples were collected in September 2021 from a wheat field and individual soil samples were obtained by mixing six randomly collected soil cores with a depth of 0–20 cm from the middle row of each plot. After homogenization, soil samples were separated into two subsamples using a 2 mm sieve. One subsample was allocated for the analysis of soil properties and microbial biomass, while the other subsample were preserved at −80°C until microbial DNA could be extracted.

### 2.2 Analyses of soil properties and microbial biomass

Soil samples were air-dried for additional analysis of other soil properties after the soil water content (SWC) was determined. The soil pH was measured in a soil suspension (soil:water, 1:2.5, w/v) made in distilled water. The soil samples were ground to < 0.50 mm, and the soil was pretreated with 6 mol L^– 1^ HCl to remove inorganic carbon. The combustion method was used to determine the amount of soil organic carbon (SOC). The C/N ratio was consistent with previous studies (Liu et al., 2020). Measurement of potential carbon mineralization (PCM) was based on CO_2_–C accumulation after capillary rewetting and a 24-h incubation period (Haney et al., 2004). Particulate organic nitrogen (PON) was analyzed using the sodium hexametaphosphate method (Liu Y. et al., 2023; Liu Z. S. et al., 2023), and the modified incubation method was used to determine potential nitrogen mineralization (PNM) concentration (Haney et al., 2004). The ammonium nitrogen (NH_4_^+^-N) and nitrate nitrogen (NO_3_^−^-N) concentrations in the extracts were measured by a modified Griess-Ilosvay method using an automated analyzer (Wang et al., 2021). Soil microbial biomass carbon (MBC) and nitrogen (MBN) were measured by the chloroform-fumigation extraction method (Wu et al., 1990).

### 2.3 DNA extraction and bioinformatic analyses of metagenomic sequencing

The total DNA was extracted from soil samples (0.5 g each) using the Fast DNA®SPIN Kit (MP Biochemicals, Solon, United States) following the manufacturer’s procedures. Concentration and purity of extracted DNA was determined with TBS-380 and NanoDrop2000, respectively. DNA extract quality was checked on 1% agarose gel. Sequencing was performed by Majorbio Bio-Pharm Technology Co., Ltd. (Shanghai, China), generating 150 bp paired-end reads at enhanced depth. Reads that aligned with human or plant genomes were excluded, and remaining sequences were trimmed using Sickle and subjected to quality filtering to ensure data integrity. Raw metagenomic reads were converted to FASTQ format, followed by multiple filtering steps: adapter removal, read trimming, and elimination of low-quality reads. Clean reads were generated using fastp on the Majorbio Cloud Platform,^1^ and high-quality reads were assembled using MEGAHIT,^2^ which made use of succinct de Bruijn graphs, retaining contigs ≥ 300 bp for downstream analysis. Open reading frames (ORFs) in each assembled contig were predicted using Prodigal. ORFs ≥ 100 bp were retrieved and translated into amino acid sequences. A non-redundant gene catalog was constructed using CD-HIT^3^ with a sequence identity of 90 and 90% coverage. High-quality reads were aligned to the non-redundant gene catalogs to calculate gene abundance with 95% identity using SOAPaligner,^4^ and gene abundance in each sample was evaluated. We utilized per-million-base transcript (TPM) mapping readings to standardize abundance values in metagenomes. The non-redundant gene set was aligned against the Comprehensive Antibiotic Resistance Database (CARD v3.0.9) using Diamond^5^ to annotate ARGs with an *e*-value cutoff of 1e^−5^. Details of the 212 identified ARGs, including gene names, types, and resistance mechanisms, are listed in Supplementary Table S1.

### 2.4 Statistical analyses

Statistical analyses were conducted using the R software platform (v4.3.2^6^). Soil properties, ARGs and their resistance mechanisms were subjected to multiple comparisons using analysis of variance (ANOVA), followed by multiple comparison of means through Tukey’s HSD test, implemented using the “multcomp” package (Hothorn et al., 2008). Principal component analysis (PCA) was performed to assess soil property distributions across a matrix of proportional parameters, visualized using the “FactoMineR” package (Lê et al., 2008). A circos plot, constructed using the “circlize” package (Gu et al., 2014), depicted the abundance of soil ARGs and their resistance mechanisms. The composition of soil ARGs was represented through principal coordinate analysis (PCoA) using the “ggplot2” package (Wickham, 2009) and multifactor ANOVA (Adonis) based on Bray-Curtis distance was performed by the “vegan” package (Dixon, 2003). The volcano plot was based on the “ropls” package (Thev, 2015) for pls-da analysis and visualized by “ggplot2” package. The Venn diagram was created with the “Venn diagram” package (Wen et al., 2023) and the “ggplot2” package on the basis of significantly differential genes screened by the volcano plot.

Redundancy analysis (RDA) with 999 permutations was conducted to examine ARG-soil property relationships, implemented in vegan. Mantel tests, also via “vegan,” assessed correlations between specific ARG types and resistance mechanisms (identified from volcano and Venn analyses) and soil properties. Random forest (RF) regression quantified the influence of soil properties on ARG abundance and resistance mechanisms. Pearson correlation coefficients between specific ARG types and soil properties were calculated and visualized using the “corrplot” package (Wei and Simko, 2024). The “Hmisc” and “igraph” packages were used to identify the potential associations (spearman’s correlation |*r*| > 0.7, *p* < 0.05) between the subtypes of soil ARGs and properties and the networks were illustrated with the collaborative platform Gephi.

## 3 Results

### 3.1 Variations in soil properties and microbial biomass under distinct crop rotation regimes

The ANOVA results indicated that most soil properties showed significant differences (*p* < 0.05) across different crop rotation regimes and complexity levels (Supplementary Table S2). Specifically, soil pH, SOC, PON, MBN, C/N ratio, and PCM/PNM differed significantly (*p* < 0.05) between the BL regimes and other crop rotation regimes. Similarly, significant differences (*p* < 0.05) in soil pH, MBN, and C/N ratio were observed between the W regimes and other crop rotation regimes (PWWM, CWWM, and A4PoW3). In comparison, the values of NO₃^−^-N, PCM/PNM, and MBN were the highest under the CWWM regime, with the PCM/PNM value almost double that under other treatments and exhibiting a significant difference (*p* < 0.05) compared with that under the A4PoW3 regime. Conversely, NH₄^+^-N and MBC showed the highest values under PWWM, with MBC being approximately twice the level observed in BL, demonstrating a significant difference (*p* < 0.05) relative to BL. Nevertheless, NO₃^−^-N and NH₄^+^-N did not show significant variations among the different rotation regimes. Furthermore, soil pH and C/N ratio were highest in BL, followed by those under non-rotation and simple rotation regimes, and lowest in the complex rotation regime.

**Figure 1:**
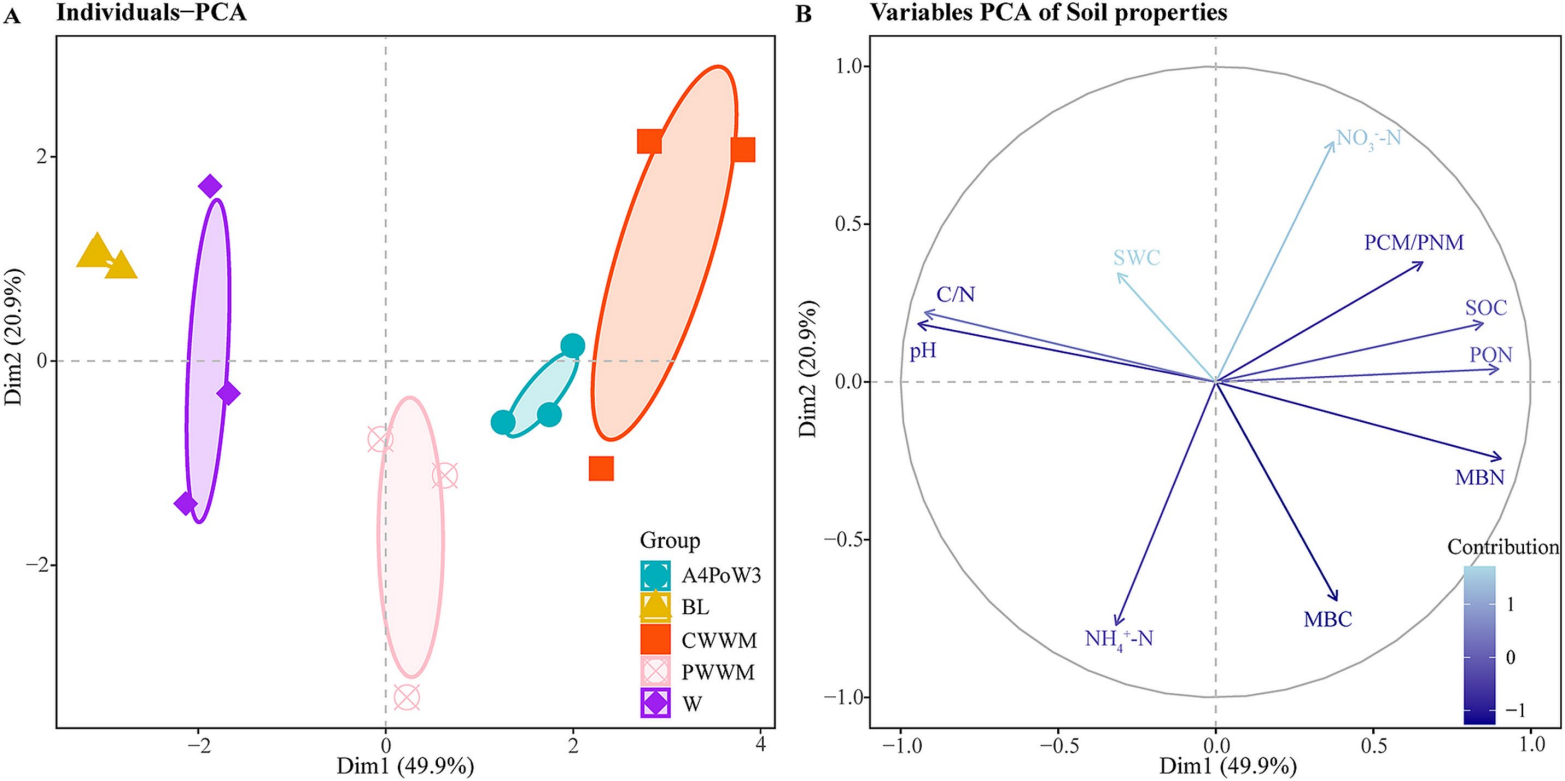
Principal component analysis (PCA) of soil properties under different crop rotation systems. **(A)** Ordinal distribution pattern. The same color indicates the same group. The horizontal axis represents the first principal component coordinate and its contribution rate, while the vertical axis represents the second principal component coordinate and its contribution rate. Ellipses denote 95% confidence intervals, with distinct ellipses corresponding to different treatment groups. **(B)** Contribution of each variable to the principal components. The depth of the arrow line color represents the degree of contribution. Darker hues indicate higher contributions. The angle of the arrow line represents the correlation. Acute angles (<90°) indicate strong positive correlations. Obtuse angles (>90°) reflect strong negative correlations. Right angles (≈90°) suggest no significant correlation. BL, bare ground; W, continuous winter wheat; PWWM, pea-winter wheat-winter wheat-wheat; CWWM, corn-winter wheat-winter wheat-wheat; A4PoW3, alfalfa-potato-winter wheat. pH, soil pH; SWC, soil water content; NH_4_^+^-N, ammonium nitrogen; NO_3_^−^-N, nitrate nitrogen; and SOC, total organic carbon; MBC, soil microbial biomass carbon; MBN, soil microbial biomass nitrogen; C/N, total carbon to nitrogen ratio; PCM/PNM, potential carbon mineralization/potential nitrogen mineralization ratio; PON, particulate organic nitrogen.

Additionally, principal component analysis (PCA) revealed that the first two principal components accounted for 70.8% of the percent variability (PC1 = 49.9% and PC2 = 20.9%) in soil properties, with the measured soil properties indicators clustered in distinct groups (Figure 1A). The contribution of each variable to the principal components varied, with their interrelationships—reflected by the angles between vectors—supported by subsequent correlation analysis. Soil pH, C/N, PON, MBN, and SOC were the dominant contributors to Dim1, while NO₃^−^-N, NH₄^+^-N, and MBC exhibited stronger associations with Dim2. Notably, the C/N ratio was negatively correlated with most soil properties, except for soil pH and soil water content (SWC). SOC was significantly positively correlated with PON and MBN (Figure 1B). Overall, soil properties displayed clear differentiation across crop rotation regimes and complexity, with their inter-dependencies varying accordingly.

### 3.2 Distribution and composition of soil microbial antibiotic resistance genes and their resistance mechanisms

**Figure 2:**
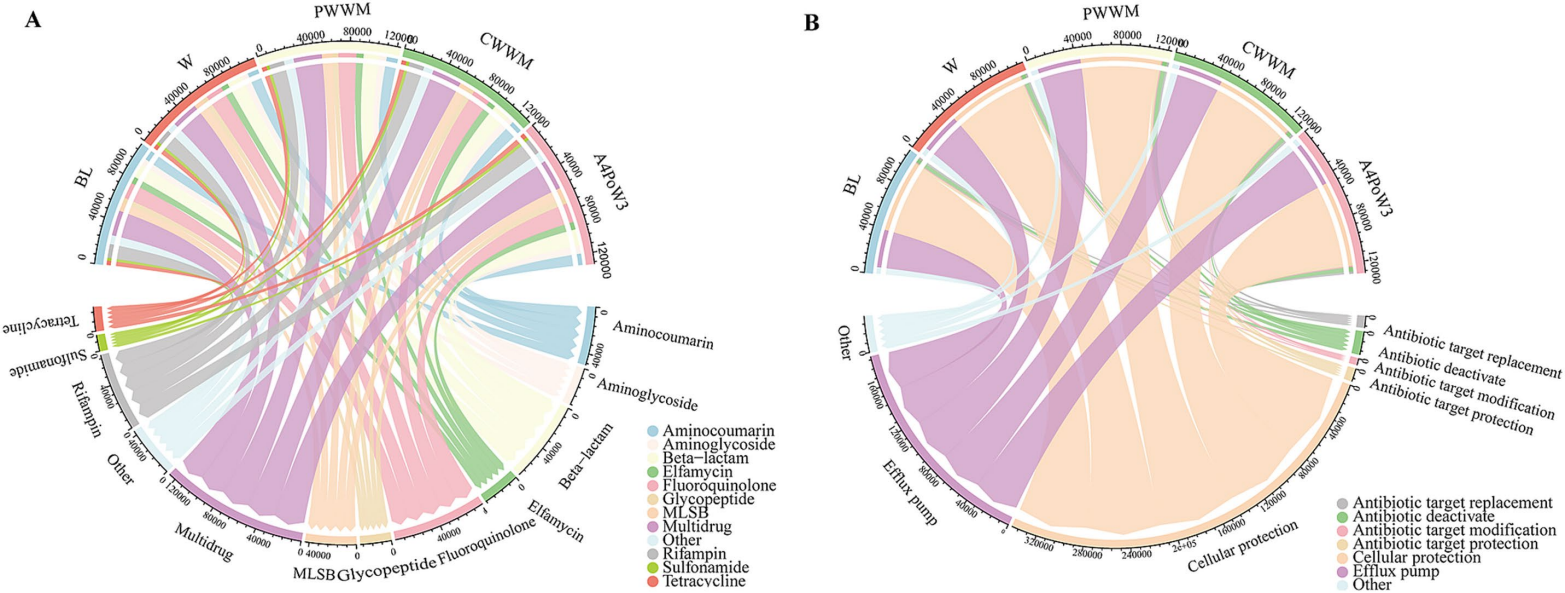
Abundance and composition of soil ARGs and their resistance mechanisms. **(A)** Circos plots showing the distribution of 12 resistance gene types under different crop rotation systems. The thickness of each ribbon represents the abundance of crop rotation regimes assigned to different resistance gene types. **(B)** Circos plots showing the distribution of 7 resistance mechanisms under different crop rotation systems. The thickness of each ribbon represents the abundance of crop rotation regimes assigned to different 7 resistance mechanisms. A4PoW3, alfalfa-potato-winter wheat. BL, bare land; CWWM, corn-winter wheat-winter wheat-millet; PWWM, pea-winter wheat-winter wheat-millet; W, continuous winter wheat.

**Figure 3:**
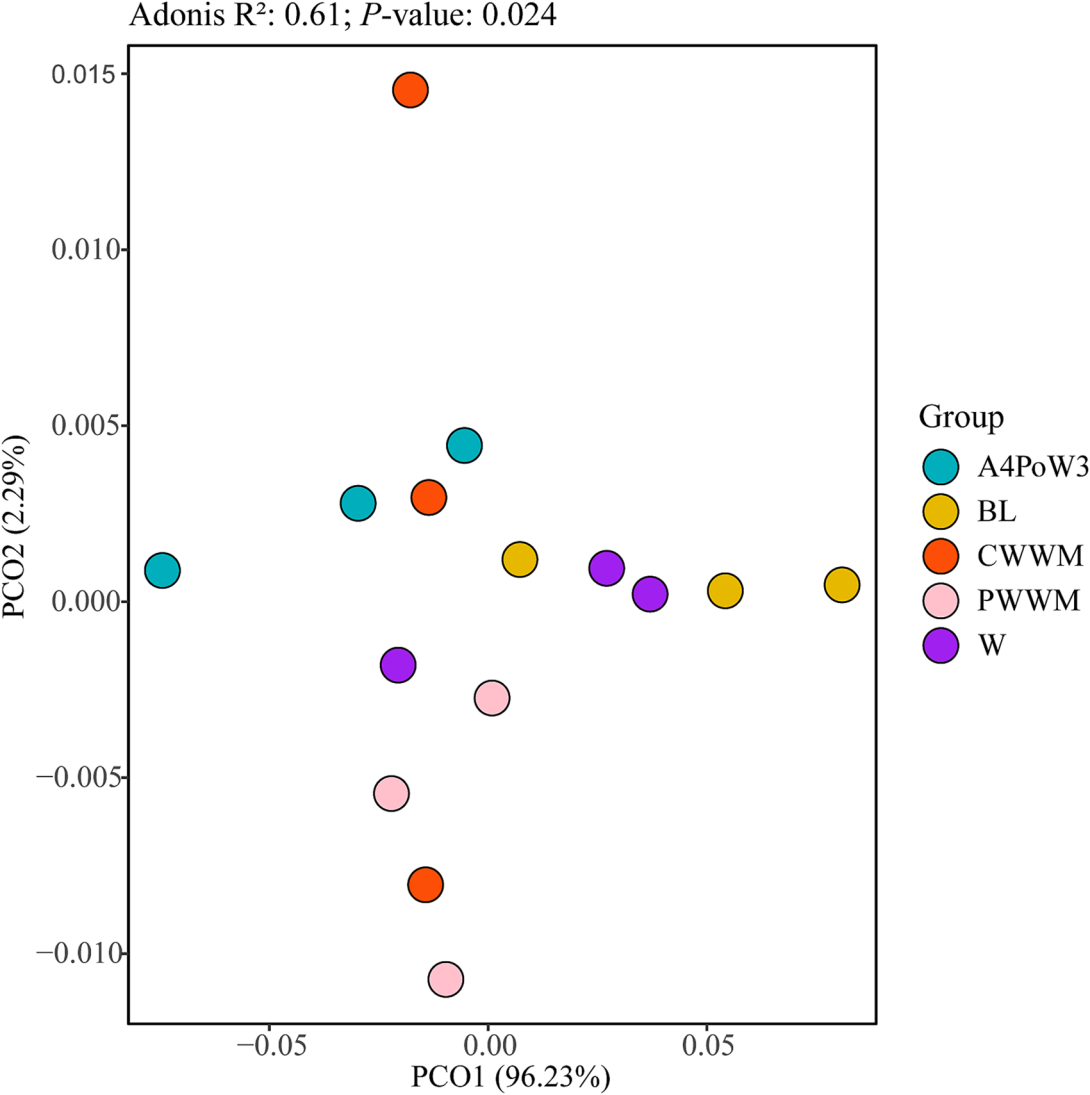
Principal Coordinate Analysis (PCoA) of ARGs under different crop rotation systems. PCOA1 and PCOA2 are two principal coordinate components. Each point in the figure represents a sample, and the color of the point represents the group of the sample. The R^2^ (ADONIS) quantifies the explained variance (higher values indicate greater explanatory power). *p* < 0.05 indicates a significant difference. A4PoW3, alfalfa-potato-winter wheat. BL, bare land; CWWM, corn-winter wheat-winter wheat-millet; PWWM, pea-winter wheat-winter wheat-millet; W, continuous winter wheat.

The diversity of soil ARG subtypes was assessed, revealing a total of 212 distinct ARG subtypes across five crop rotation regimes (Supplementary Table S3). These ARG subtypes were classified into 12 resistance categories, including aminocoumarin, aminoglycoside, beta-lactam, elfamycin, fluoroquinolone, glycopeptide, MLSB, multidrug, rifampin, sulfonamide, tetracycline, and others (Supplementary Table S4). The abundance of soil ARGs exhibited significant variations (*p* < 0.05) among different crop rotation regimes (Figure 2A). Notably, fluoroquinolone and multidrug resistance genes were the most abundant ARGs across all regimes examined (Supplementary Figure S1). To investigate the effect of different crop rotation regimes and complexity on the distribution of soil ARGs, PCoA was conducted using the abundance data of soil ARGs (Figure 3). The PCoA revealed distinct separation among the A4PoW3, PWWM, and BL regimes along the axes, indicating that crop rotation complexity significantly shapes the composition of soil ARGs. Furthermore, ADONIS analysis (*R*^2^ = 0.61, *p* < 0.05) confirmed the statistical significance of this separation. These findings demonstrate that crop rotation regimes significantly affected the distribution and composition of soil ARGs.

Compared to BL, the number of significantly differential genes (*p* < 0.05) varied across treatments: W had 11 genes, PWWM had 18 genes, CWWM had 15 genes, and A4PoW3 had 30 genes (Supplementary Figure S2). This indicated that various treatments had distinct impacts on ARGs. In the A4PoW3 regime, differentially abundant genes were most numerous, with a higher count of specific ARG subtypes compared to other regimes (Supplementary Table S5), with the largest number of ARGs in the glycopeptide and multidrug categories, followed by those in the fluoroquinolone category. Some differential genes, such as oleR and oleB (both MLSB), were consistently observed across distinct contrast groups, including W vs. BL, PWWM vs. BL, CWWM vs. BL, and A4PoW3 vs. BL (Supplementary Figure S3).

In this study, resistance mechanisms under different crop rotation regimes were analyzed, including antibiotic target replacement, antibiotic deactivation, antibiotic target modification, antibiotic target protection, cellular protection, and efflux pump. The results showed that cellular protection and efflux pump were the two predominant resistance mechanisms across all crop rotation regimes (Figure 2B) and the abundance of soil ARGs associated with these resistance mechanisms showed significant differences among the different treatment groups (*p* < 0.05; Supplementary Table S6). In A4PoW3, the abundance of genes related to cellular protection and efflux pump was significantly higher than that in other treatments, with multidrug resistance genes particularly enriched in the efflux pump mechanism (Supplementary Table S1).

### 3.3 Correlations between soil properties, microbial biomass, and antibiotic resistance genes and their resistance mechanisms

The RDA was employed to assess the influence of soil properties and microbial biomass on ARG abundance (Supplementary Figure S4). The results indicated that C/N ratio, pH, MBC, and MBN can significantly explain the variation in the abundance of resistance genes, and their effects on the abundance of soil ARGs were statistically significant (Supplementary Table S7). MBC, MBN, and SOC were significantly correlated with soil ARGs under A4PoW3, and the correlation between soil C/N ratio and pH and soil ARGs was particularly significant under BL. Our findings reveal that differential crop rotation regimes and complexity potentially modify edaphic factors, which are significantly associated with ARG abundance and dispersal characteristics in soils.

**Figure 4:**
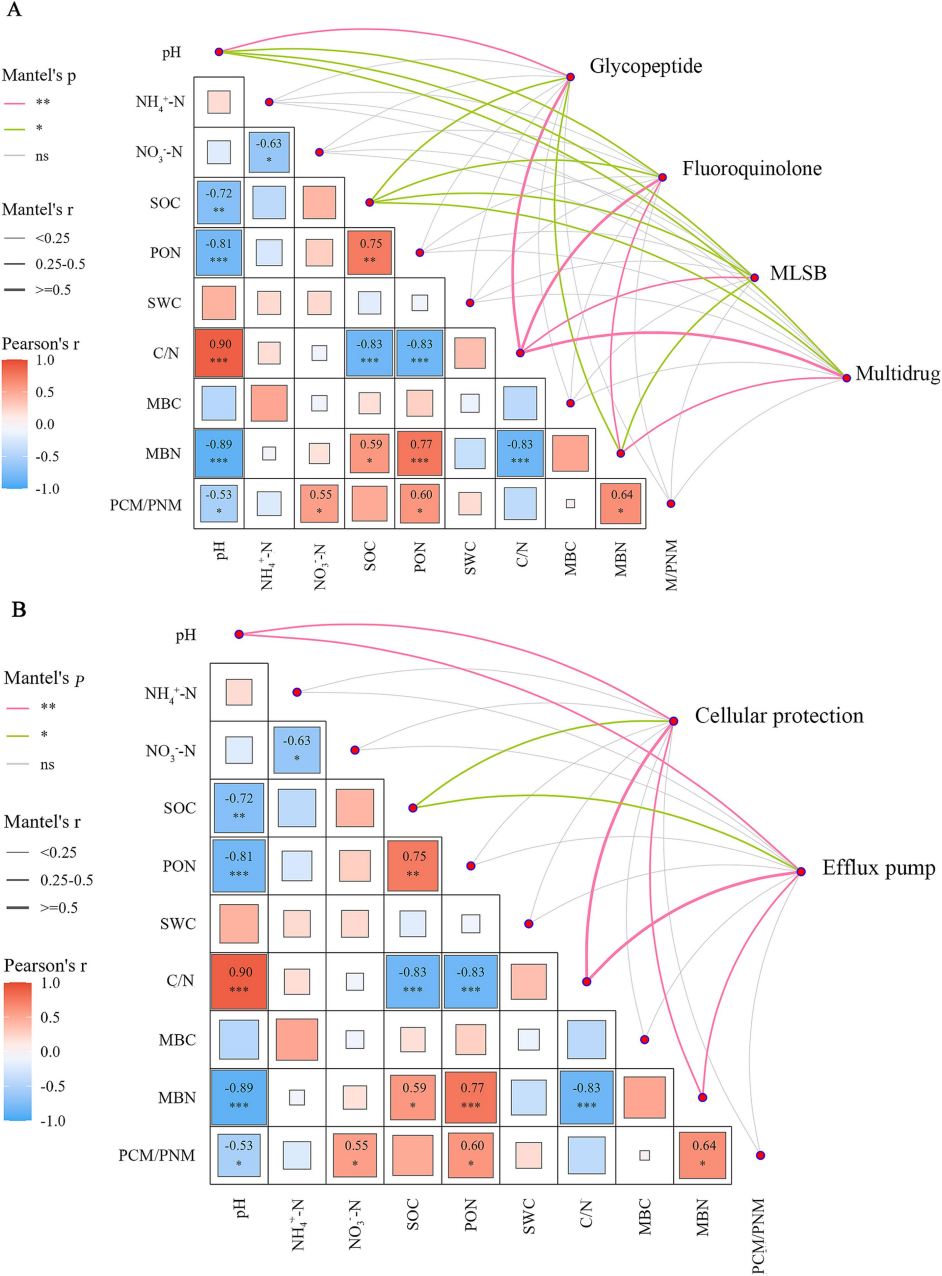
Relationship between soil properties and ARGs and their resistance mechanisms. **(A)** Correlation analysis between soil properties and ARGs based on Mantel test. Mantel r and *p* values (*p < 0.05; **p < 0.01), as well as Pearson *r* values, are indicated by the color and width of the connection lines as specified in the legend. **(B)** Correlation analysis between soil properties and resistance mechanisms based on Mantel test. Mantel r and *p* values (*p < 0.05; **p < 0.01), as well as Pearson *r* values, are indicated by the color and width of the connection lines as specified in the legend. pH, soil pH; SWC, soil water content; NH_4_^+^-N, ammonium nitrogen; NO_3_^−^-N, nitrate nitrogen; and SOC, total organic carbon; MBC, soil microbial biomass carbon; MBN, soil microbial biomass nitrogen; C/N, total carbon to nitrogen ratio; PCM/PNM, potential carbon mineralization/potential nitrogen mineralization ratio; PON, particulate organic nitrogen.

A Mantel test (Supplementary Tables S8, S9) was conducted to examine the correlation between specific ARG types (glycopeptide, multidrug, fluoroquinolones, and MLSB) and resistance mechanisms (efflux pump and cellular protection) with various soil properties and microbial biomass—soil pH, SWC, NH_4_^+^-N, NO_3_^−^-N, SOC, MBC, MBN, C/N ratio, and PCM/PNM—with analyses performed across different crop rotation regimes. The results revealed soil pH, SOC, C/N ratio, and MBN were significantly correlated with specific ARG types and resistance mechanisms (*p* < 0.05; Figure 4). Notably, of these soil properties, soil C/N consistently explained the highest proportion of variance in specific ARG types and resistance mechanisms, which was further confirmed by the RF analysis (Tables 1, 2). In crop rotation regimes, soil properties and microbial biomass were identified in ARG interaction networks (Supplementary Figure S5). C/N ratio recorded the highest node connectivity in the ARG network (node degree = 88; Supplementary Table S10), indicating the crucial role of the C/N ratio in maintaining the stability of the resistance gene network. Overall, the complexity of different crop rotation regimes, particularly their impact on soil C/N ratio, may be associated with the abundance and distribution of ARG types and resistance mechanisms.

**Table 1:** Random forest mean predictor importance of the soil properties for the abundance of the representative types of antibiotic resistance genes.

Fluoroquinolone (*R^2^* = 47.7%, *p* < 0.001)			Glycopeptide (*R*^2^ = 13.2%, *p* > 0.05)			MLSB (*R*^2^ = 24.3%, *p* < 0.05)			Multidrug (*R*^2^ = 37.6%, *p* < 0.05)		
Soil properties	%IncMSE	%IncMSE.*p*	Soil properties	%IncMSE	%IncMSE.*p*	Soil properties	%IncMSE	%IncMSE.*p*	Soil properties	%IncMSE	%IncMSE.*p*
**C/N**	9.691	0.010	**C/N**	6.292	0.020	**C/N**	7.758	0.010	**C/N**	8.253	0.010
MBN	7.560	0.010	MBC	4.257	0.040	MBC	5.701	0.010	MBC	5.166	0.030
MBC	6.254	0.010	PON	5.107	0.050	pH	5.131	0.030			
PON	6.062	0.010									
pH	4.425	0.030									

pH, soil pH; MBC, soil microbial biomass carbon; MBN, soil microbial biomass nitrogen; C/N, total carbon/nitrogen ratio; PON, particulate organic nitrogen. The exhibition soil properties indicate significant effects (*p* < 0.05). The fitting degree (R^2^) and the significance (*p*-values) of the random forest regression model are provided.

**Table 2:** Random forest mean predictor importance of the soil properties for the abundance of the representative types of antibiotic resistance mechanisms.

Cellular protection (*R*^2^ = 38.7%, *p* < 0.001)			Efflux pump (*R*^2^ = 39.2%, *P* < 0.001)		
Soil properties	%IncMSE	%IncMSE.pval	Soil properties	%IncMSE	%IncMSE.pval
**C/N**	9.028	0.010	**C/N**	8.672	0.020
MBN	5.568	0.040	MBC	4.781	0.020
MBC	4.418	0.030	MBN	6.145	0.020
PON	5.544	0.040	pH	5.133	0.040
SOC	5.429	0.050			

pH, soil pH; SOC, total organic carbon; MBC, soil microbial biomass carbon; MBN, soil microbial biomass nitrogen; C/N, total carbon/nitrogen ratio; PON, particulate organic nitrogen. The exhibition soil properties indicate significant effects (*P* < 0.05). The fitting degree (R^2^) and the significance (*P*-values) of the random forest regression model are provided.

## 4 Discussion

### 4.1 Effects of crop rotation regime and complexity on soil properties and microbial biomass

Crop rotation plays a critical role in enhancing soil fertility (Giacometti et al., 2021). Crop rotation effectively improves the chemical and biological characteristics of soil through mechanisms such as plant root growth, rhizosphere microbial activity, and the accumulation of organic matter (McDaniel and Grandy, 2016; Giacometti et al., 2021). Crop rotation regime and complexity significantly enhanced soil microbial activity and nitrogen mineralization. We found that PCM/PNM (9.95 ± 0.68) and MBN (63.83 ± 1.19) in the CWWM regime were significantly higher values than in other treatment regimes and MBC value was the highest in the PWWM group. This phenomenon may be attributed to maize, a carbon-rich and nitrogen-demanding crop. When integrated into diversified rotations, maize enhances microbial activity and accelerates organic matter decomposition, thereby promoting nitrogen mineralization (McDaniel and Grandy, 2016). Additionally, the input of high C/N ratio residues (such as corn) drives microorganisms to prioritize the mineralization of nitrogen-containing compounds to meet their nitrogen demands and maintain a stable C/N ratio in crop rotation systems. This process suppresses the secretion of carbon-degrading enzymes, delaying carbon mineralization. Consequently, the difference in carbon and nitrogen mineralization rates becomes more pronounced, leading to a faster accumulation of PCM compared to PNM (Islam et al., 2022; Jiang et al., 2025). The introduction of leguminous plants enhances soil microbial activity by providing additional nitrogen sources and organic matter, thereby increasing the soil MBC content. This discovery provides direct evidence that legume crop rotation improves soil fertility. Although the contents of NH_4_^+^-N and NO_3_^−^-N varied across different cropping systems, these differences were not significant (*p* > 0.05). The increase in soil temperature and water content enhanced the mineralization of soil organic nitrogen (Fu et al., 2019). Combined with the lack of nitrogen uptake by crops, this leads to an increase in NH_4_^+^-N levels (Fu et al., 2019). Additionally, the application of legume residues and nitrogen fertilizer can also influence the NH_4_^+^-N content (Hu et al., 2023). Soil NO_3_^−^-N is lost through runoff and leaching, and high nitrogen fertilizer input affects NO_3_^−^-N levels as well (Fu et al., 2019; Hu et al., 2023). Overall, the variations in NH_4_^+^-N and NO_3_^−^-N were regulated by multiple factors, and the effects of different cropping systems were offset, resulting in no significant differences. Furthermore, soil pH were the highest in BL and the lowest in A4PoW3. Compared to the BL treatment, the A4PoW3 treatment introduced more diverse crops, and the root exudates of these crops mostly possessed acidic properties (Lv et al., 2023). The roots may have lowered the soil pH by increasing the release of acidic compounds, leading to differences in pH across the different crop rotations (Wen et al., 2017). Furthermore, changes in soil pH altered nutrient availability, ultimately affecting soil fertility and its capacity to support crop growth. In this study, long-term diversified crop rotation reduced the C/N ratio. Previous studies have shown that introducing legumes into long-term diversified planting may effect nitrogen balance, but it may not have a significant impact on carbon (Islam et al., 2022).

Additionally, different crop rotation regimes affect the soil environmental condition through the input of post-harvest crop residues and various root exudates secreted during the different growth stages of plants (Crotty et al., 2016), leading to the significant differences in individual physicochemical properties observed in the PCA. Moreover, strong interdependencies observed between the C/N ratio, other soil properties, and microbial biomass. C/N ratio influences the decomposition rate of organic matter, microbial community activity, and nutrient release and fixation, thereby affecting soil structural stability, nutrient availability, and pH buffering capacity (Wang et al., 2024).

### 4.2 Effects of crop rotation regimes on the dynamics of antibiotic resistance genes and their resistance mechanisms

Agricultural management practices are closely associated with the contamination of soil with microbial ARGs (Pruden et al., 2013). In this investigation, fluoroquinolone and multidrug resistance genes emerged as the most abundant ARG types. The widespread use of fluoroquinolones and multidrug antibiotics in agriculture (Sun et al., 2017; Huang et al., 2021; Wu et al., 2023; Zhang, 2024) has led to the adaptation of microbial communities to antibiotic selection pressure (Forsberg et al., 2014; Jechalke et al., 2014). This adaptation process leads to the selective enrichment and persistence of corresponding resistance genes in soil. Additionally, under diversified crop rotation systems, there are complex interactions between root exudates and microorganisms in the rhizosphere soil (Mishra et al., 2022). These interactions may promote the faster proliferation and transmission of some ARGs in the soil (Haichar et al., 2008). Fertilization reshapes the structure of rhizosphere microbial communities, driving the adaptive evolution of environmental microorganisms toward a higher resistance gene profile (Guan et al., 2025). These changes promote the expression and transmission of ARGs (Gao et al., 2023; Wang et al., 2023), which may lead to increased resistant microorganisms and ARG accumulation. The PCoA results showed a significant separation of ARGs among crop rotation regimes with varying complexity. Crop rotation indirectly shapes ARG diversity and abundance by changing the structure of soil microbial communities (Zhang et al., 2023) and affecting stability of their ecological networks (Fan et al., 2022).

Furthermore, different crop rotation regimes significantly affected the abundance of soil ARGs (*p* < 0.05). The enrichment of agricultural soil ARGs is influenced by the cultivation of different crop types (Cerqueira et al., 2019; Huang et al., 2021). Crop root exudates play an important role in this process by regulating the structure and function of rhizosphere microbial communities, indirectly promoting the growth of ARGs in the soil–plant system (Gao et al., 2023). In A4PoW3, the number of differential genes was the highest among all treatments. This likely resulted from the use of a more complex crop combination and extended cultivation duration, which resulted in richer and more complex root exudates and crop residue inputs (Bowles et al., 2022). These inputs may provide diverse carbon and nitrogen sources for the soil microbial communities, thereby enhancing microbial activity and metabolic process diversity. Such stimulation of microbial functions could significantly influence distribution patterns of ARGs. This phenomenon highlights the vital role of crop diversity in regulating the distribution of soil ARGs (Cerqueira et al., 2019). Additionally, differences in *oleR* and *oleB* across regimes indicates the transfer of relevant potential genes in the microbial community and exposure to selection pressure under different crop rotation regimes. This finding further emphasizes the dynamic role of microbial communities in the spread of soil ARGs (Zhang et al., 2022).

Our findings indicate that efflux pumps and cellular protection were significant resistance mechanisms under crop rotation. The resistance mechanisms efflux pump and cellular protection impart resistance to various antibiotics. Efflux pumps expel various types of antibiotic molecules from the cells (Zack et al., 2024), and the cellular protection mechanism protects numerous target proteins and nucleic acids from antibiotics (Darby et al., 2023). Efflux pumps and cellular protection were identified as the main resistance mechanisms (Alshehri et al., 2023) in the root nodule soils associated with the naturally growing *Abutilon fruticosum*. Root secretions of different crops affect the soil microbial communities under different crop rotation regimes (Li et al., 2025). In microbial communities, different resistance mechanisms are selected for through multiple interactions (Bottery et al., 2021), which increases the soil microbial antibiotic resistance (Xiao et al., 2023).

### 4.3 Contribution of soil properties and microbial biomass to soil microbial antibiotic resistance genes

Both RDA and the Mantel test results indicated that soil properties and microbial biomass were significantly correlated with the abundance and distribution of soil ARGs (*p* < 0.05). Soil properties and microbial biomass may impact the abundance and distribution of soil ARGs by affecting the transmission of soil ARGs regulated by the composition and species diversity of soil microbial communities (Wu et al., 2023). In this study, MBC, MBN, and SOC showed a more significant correlation with the distribution of soil ARGs under the A4PoW3 treatment, suggesting that the relationship between microbial communities and ARGs is more pronounced under a complex crop rotation regime. Soil pH, SOC, MBN, C/N were found to be significantly correlated with ARGs types as well as their resistance mechanisms (*p* < 0.05). Soil pH emerged as a critical driver of microbial community evolution. In general, bacteria were most abundant in neutral environments, and soil pH influences microbial selection by altering nutrient availability or affecting physiological activity. Certain pH values might apply direct pressure on bacterial cells and lead to the selection of certain bacterial populations, indirectly affecting the production of ARGs (Han et al., 2022). SOC serves as a carbon and nutrient source for bacteria and influence the abundance of soil ARGs by promoting the growth of ARG-harboring bacteria (Guo et al., 2020; Shen et al., 2023). Through further network analysis and prediction using random forests, identified that the C/N ratio might be the most relevant factor. The C/N ratio directly affects microbial nutritional status and metabolic efficiency (Gaudel et al., 2024). According to the stoichiometric decomposition theory (Chen et al., 2014), microorganisms tend to consume resources that meet their stoichiometric requirements to maintain their ideal elemental stoichiometric ratios. The C/N ratio may influence the activity and function of microbial communities by regulating microbial energy metabolism. The C/N ratio not only regulates microbial metabolic activities but may also further alter the efficiency of horizontal gene transfer by affecting resource utilization efficiency and microbial community structure. It enhances the ecological adaptability of certain microorganisms with competitive advantages in resource acquisition (Ding and Sun, 2025), promoting the gene exchange efficiency among microorganisms carrying specific resistance genes (Shen et al., 2023). In summary, the C/N ratio significantly affects microbial community structure and its ecological functions by regulating microbial energy metabolism, horizontal gene transfer efficiency, and carbon/nutrient competition. These mechanisms collectively explain why the C/N ratio becomes a key factor related to the distribution of ARGs.

In this study, the correlation between specific ARG types and soil properties as well as microbial biomass was analyzed using the Mantel test. Soil pH, SOC, MBN, C/N ratio were found to be significantly correlated with ARGs types as well as their resistance mechanisms (*p* < 0.05). These four factors may exhibited selective effects on specific ARG types. For example, they influences ARGs by promoting the growth of bacteria carrying specific ARGs (Zhang et al., 2024), shaping the resistome (Liu Z. S. et al., 2023), and enhancing the competitive advantage of specific ARG-carrying bacteria (Zheng et al., 2022). Future research could further explore the mechanisms of changes in microbial metabolic pathways under different C/N ratios, and how these changes further regulate soil functions and the spread of resistance genes by affecting microbial community dynamics and gene exchange efficiency.

## 5 Conclusion

In this study, we examined the effects of crop rotation regimes and complexity on soil properties, microbial biomass, and the distribution, composition, and resistance mechanisms of soil ARGs using metagenomics and multivariate statistical analyses. Our findings demonstrated that crop rotation regimes and complexity significantly influenced most soil properties and microbial biomass, resulting in distinct separation and significant differences among regimes. Similarly, the abundance and distribution of soil ARGs exhibited significant variation across crop regimes, reflecting the impact of rotation complexity. Furthermore, analysis of differently abundant genes further enabled the identification of specific ARG types and their associated resistance mechanisms. The results suggest that soil properties are correlated with the selection of ARGs and their resistance mechanisms, with the C/N ratio being the most significant correlated factor. Collectively, these findings offer a comprehensive insight into the changes in soil ARGs under long-term crop rotation regimes, highlighting the interplay between long-term crop rotation systems, soil environmental conditions, and microbial resistance.

## Data Availability

The datasets presented in this study can be found in online repositories. The names of the repository/repositories and accession number(s) can be found at: https://www.ncbi.nlm.nih.gov/sra/PRJNA985043.

## Footnotes

^1^ https://github.com/OpenGene/fastp

^2^ https://github.com/voutcn/megahit

^3^ http://www.bioinformatics.org/cd-hit/

^4^http://soap.genomics.org.cn/, version 2.21.

^5^http://www.diamondsearch.org/index.php, version 0.8.35.

^6^ http://www.r-project.org/
