# Supplementary material for: Crop rotation complexity affects soil properties shaping antibiotic resistance gene types and resistance mechanisms
Source: Front Microbiol. 2025 Jun 25;16:1603518. doi: 10.3389/fmicb.2025.1603518 (PMC12237906; doi:10.3389/fmicb.2025.1603518)
Supplement: Supplementary file 1 [file Data_Sheet_1.docx]

Supplementary information includes ten tables, four figures, and additional details about the field experiment design.

**Summary**

**Table S1** The antibiotic resistance ontology, name, resistance mechanism and description of antibiotic resistance genes referring to the CARD database.

**Table S2** Soil properties and microbial biomass among distinct crop rotational regimes and complexity (n = 3); values are mean ± standard deviation.

**Table S3** The abundance of antibiotic resistance genes at subtype level among distinct crop rotational regimes (n = 3). Values are mean ± standard deviation.

**Table S4** The abundance of antibiotic resistance genes among distinct crop rotational regimes (n =3). Values are mean ± standard deviation.

**Table S5** The number of ARG subtypes among distinct contrast group (n = 3).

**Table S6**The representative resistance mechanisms among distinct crop rotational regimes (n = 3); values are mean ± standard deviation.

**Table S7** Correlations among the representative ARGs and the soil properties determined using the Mantel test.

**Table S8** Correlations among the representative antibiotic resistance mechanisms and the soil properties determined using the Mantel test.

**Table S9** Environmental factors in RDA significantly affect ARGs based on 999 permutation test.

**Table S10** Node degree of soil properties and microbial biomass in the network.

**Fig. S1** Relative abundance of ARGs types across distinct crop rotation regimes.

**Fig. S2** Volcano plot of differential resistance genes under different crop rotation systems.

**Fig. S3** Veen plots of differential genes in different comparison groups.

**Fig. S4** Redundancy analysis (RDA) between ARGs and soil properties under different crop rotation systems.

**Fig. S5** Networks between soil properties and microbial biomass and ARGs.

**Additional details about the field experiment design**

The experiment was conducted using a randomized complete block design with three replications. Each individual plot measured 10.3 m × 6.5 m, with a 0.5-m spacing between plots and a 1-m buffer strip separating the blocks. Five crop rotations, with rotation lengths ranging from 1 to 10 years, were selected for this study. All crops were hand-planted under a conventional tillage regime. For the first 16 years, soil preparation was carried out using animal-drawn plows, while in the subsequent 14 years, a hand tractor-drawn plow was utilized, achieving a tillage depth of 10 cm at planting.

The planting densities for winter wheat, pea, corn, and potato were 2.23, 0.60, 0.04, and 0.5 million plants ha⁻¹, respectively. Millet and alfalfa were sown at rates of 28 kg ha⁻¹ and 9 kg ha⁻¹, respectively. Crops were planted in rows spaced 20 cm apart, with the exception of corn and potato, which were planted at a row spacing of 70 cm.

Fertilization included nitrogen (N) and phosphorus (P) applications: winter wheat, corn, millet, and potato received urea (46% N) and monoammonium phosphate (11% N, 23% P) at a rate of 120 kg N ha⁻¹, and P was applied at 62 kg P ha⁻¹ using monoammonium phosphate. Pea and alfalfa received phosphorus fertilizer at the same rate (62 kg P ha⁻¹) as well as nitrogen at a lower rate of 7 kg N ha⁻¹. Potassium (K) fertilizer was not applied because the soil was naturally enriched with sufficient levels of K.

Weeding was conducted manually across all treatment plots, including the fallow treatment, to remove above and belowground weed biomass before, during, and after crop growth. Pesticides were applied as needed to manage pests. All crops were cultivated under rainfed conditions, with no supplemental irrigation provided.

Description of crop rotations:

BL: No crops were planted.

W: Wheat was planted in late September and harvested in late June each year.

PWWM: Peas were planted in mid-March and harvested in early July. Winter wheat was then planted in late September and harvested in early June of the following year. This process was repeated with winter wheat being planted again in September and harvested in early June of the third year. Millet was planted in late June and harvested in mid-September.

CWWM: Corn was planted in mid-April and harvested in mid-September. Winter wheat was planted in late September and harvested in early June of the following year. This cycle continued with winter wheat being planted again in September and harvested in early June of the third year. Millet was planted in late June and harvested in mid-September.

A4PoW3: Alfalfa was planted in March and terminated in February for the first four years. Alfalfa biomass was harvested two to three times each year, depending on growth. In the fifth year, potatoes were planted in early March and harvested in mid-September. Winter wheat was planted in late September and harvested in early June for the next three years.

We explicitly defined crop rotation complexity using a quantitative Rotation Complexity Index (RCI) (reference: Long-term evidence shows that crop-rotation diversification increases agricultural resilience to adverse growing conditions in north America), calculated as:

$$RCI=\sqrt{(Number of crop species)\times(Rotation length in years)}$$

This index integrates both crop species richness (crop species richness) and crop cover duration (crop cover duration).

The RCI values were categorized as:

Non-rotation (W): RCI = 1 (single crop, 1-year cycle)

Simple rotations (PWWM, CWWM): RCI = 3 (3 species, 3-year cycle)

Complex rotation (A4PoW3): RCI = 5 (3 species, 8-year cycle)

**Table 1** Random Forest mean predictor importance of the soil properties for the abundance of the representative types of antibiotic resistance genes.

| Fluoroquinolone（R^2^ = 47.7%, *P* < 0.001） | | | Glycopeptide (R^2^ = 13.2%, *P* > 0.05) | | | MLSB (R^2^ = 24.3 %, *P* < 0.05) | | | Multidrug (R^2^ = 37.6%, *P* < 0.05) | | |
| --- | --- | --- | --- | --- | --- | --- | --- | --- | --- | --- | --- |
| Soil properties | %IncMSE | %IncMSE.*p* | Soil properties | %IncMSE | %IncMSE.*p* | Soil properties | %IncMSE | %IncMSE.*p* | Soil properties | %IncMSE | %IncMSE.*p* |
| C/N | 9.691 | 0.010 | C/N | 6.292 | 0.020 | C/N | 7.758 | 0.010 | C/N | 8.253 | 0.010 |
| MBN | 7.560 | 0.010 | MBC | 4.257 | 0.040 | MBC | 5.701 | 0.010 | MBC | 5.166 | 0.030 |
| MBC | 6.254 | 0.010 | PON | 5.107 | 0.050 | pH | 5.131 | 0.030 |  |  |  |
| PON | 6.062 | 0.010 |  |  |  |  |  |  |  |  |  |
| pH | 4.425 | 0.030 |  |  |  |  |  |  |  |  |  |

Note: pH, soil pH; MBC, soil microbial biomass carbon; MBN, soil microbial biomass nitrogen; C/N, total carbon/nitrogen ratio; PON, particulate organic nitrogen. The exhibition soil properties indicate significant effects (*P* < 0.05). The fitting degree (R^2^) and the significance (*P-values*) of the random forest regression model are provided.

**Table 2** Random Forest mean predictor importance of the soil properties for the abundance of the representative types of antibiotic resistance mechanisms.

| Cellular protection（R^2^ = 38.7%,*P* < 0.001） | | | Efflux pump (R^2^ = 39.2%,*P* < 0.001) | | |  |
| --- | --- | --- | --- | --- | --- | --- |
| Soil properties | %IncMSE | %IncMSE.pval | Soil properties | %IncMSE | %IncMSE.pval | |
| C/N | 9.028 | 0.010 | C/N | 8.672 | 0.020 | |
| MBN | 5.568 | 0.040 | MBC | 4.781 | 0.020 | |
| MBC | 4.418 | 0.030 | MBN | 6.145 | 0.020 | |
| PON | 5.544 | 0.040 | pH | 5.133 | 0.040 | |
| SOC | 5.429 | 0.050 |  |  |  | |

Note: pH, soil pH; SOC, total organic carbon; MBC, soil microbial biomass carbon; MBN, soil microbial biomass nitrogen; C/N, total carbon/nitrogen ratio; PON, particulate organic nitrogen. The exhibition soil properties indicate significant effects (*P* < 0.05). The fitting degree (R^2^) and the significance (*P*-values) of the random forest regression model are provided.

**Table S1** The antibiotic resistance ontology, name, resistance mechanism and description of antibiotic resistance genes referring to the CARD database.

| **ARO number** | **ARG subtype** | **ARG type** | **Resistance mechanism** |
| --- | --- | --- | --- |
| 3003035 | mfpA | Other | Antibiotic target replacement |
| 3000412 | sul2 | Sulfonamide | Antibiotic target replacement |
| 3002857 | dfrA26 | Sulfonamide | Antibiotic target replacement |
| 3002875 | dfrE | Sulfonamide | Antibiotic target replacement |
| 3003105 | dfrA3 | Sulfonamide | Antibiotic target replacement |
| 3002891 | otrA | Tetracycline | Antibiotic target replacement |
| 3003930 | rpsJ | Tetracycline | Antibiotic target replacement |
| 3003677 | AAC(6')-Iaj | Aminoglycoside | Antibiotic deactivate |
| 3002524 | AAC(2')-Ib | Aminoglycoside | Antibiotic deactivate |
| 3002525 | AAC(2')-Ic | Aminoglycoside | Antibiotic deactivate |
| 3002531 | AAC(3)-Ic | Aminoglycoside | Antibiotic deactivate |
| 3002539 | AAC(3)-IV | Aminoglycoside | Antibiotic deactivate |
| 3002541 | AAC(3)-VIIa | Aminoglycoside | Antibiotic deactivate |
| 3002542 | AAC(3)-VIIIa | Aminoglycoside | Antibiotic deactivate |
| 3002593 | AAC(6')-Ib-SK | Aminoglycoside | Antibiotic deactivate |
| 3002630 | ANT(9)-Ia | Aminoglycoside | Antibiotic deactivate |
| 3002657 | APH(6)-Ia | Aminoglycoside | Antibiotic deactivate |
| 3001308 | VgbB | Aminoglycoside | Antibiotic deactivate |
| 3003744 | vatF | Aminoglycoside | Antibiotic deactivate |
| 3002662 | APH(9)-Ia | Aminoglycoside | Antibiotic deactivate |
| 3002659 | APH(6)-Ic | Aminoglycoside | Antibiotic deactivate |
| 3002544 | AAC(3)-Xa | Aminoglycoside | Antibiotic deactivate |
| 3002553 | AAC(6')-If | Aminoglycoside | Antibiotic deactivate |
| 3002556 | AAC(6')-Ii | Aminoglycoside | Antibiotic deactivate |
| 3002563 | AAC(6')-Isa | Aminoglycoside | Antibiotic deactivate |
| 3000606 | FEZ-1 beta-lactamase | Beta-lactam | Antibiotic deactivate |
| 3000856 | BJP-1 | Beta-lactam | Antibiotic deactivate |
| 3002482 | LRA-1 | Beta-lactam | Antibiotic deactivate |
| 3002483 | LRA-5 | Beta-lactam | Antibiotic deactivate |
| 3002512 | LRA-17 | Beta-lactam | Antibiotic deactivate |
| 3002513 | LRA-19 | Beta-lactam | Antibiotic deactivate |
| 3003665 | NmcR | Beta-lactam | Antibiotic deactivate |
| 3003720 | SPG-1 | Beta-lactam | Antibiotic deactivate |
| 3003894 | Rm3 beta-lactamase | Beta-lactam | Antibiotic deactivate |
| 3002700 | cmlv | Other | Antibiotic deactivate |
| 3002839 | lnuF | Other | Antibiotic deactivate |
| 3000444 | rifampin phosphotransferase | Rifampin | Antibiotic deactivate |
| 3002846 | arr-1 | Rifampin | Antibiotic deactivate |
| 3002849 | arr-4 | Rifampin | Antibiotic deactivate |
| 3002883 | rgt1438 | Rifampin | Antibiotic deactivate |
| 3002884 | iri | Rifampin | Antibiotic deactivate |
| 3002814 | clbA | Other | Antibiotic target modification |
| 3002815 | clbB | Other | Antibiotic target modification |
| 3002816 | clbC | Other | Antibiotic target modification |
| 3003441 | cfrA | Other | Antibiotic target modification |
| 3003907 | CIPa | Other | Antibiotic target modification |
| 3003440 | mecB | Beta-lactam | Antibiotic target protection |
| 3000617 | mecA | Beta-lactam | Antibiotic target protection |
| 3000190 | tetO | Tetracycline | Antibiotic target protection |
| 3000191 | tetQ | Tetracycline | Antibiotic target protection |
| 3000192 | tetS | Tetracycline | Antibiotic target protection |
| 3000193 | tetT | Tetracycline | Antibiotic target protection |
| 3000194 | tetW | Tetracycline | Antibiotic target protection |
| 3000195 | tetB(P) | Tetracycline | Antibiotic target protection |
| 3000196 | tet32 | Tetracycline | Antibiotic target protection |
| 3000197 | tet36 | Tetracycline | Antibiotic target protection |
| 3000556 | tet44 | Tetracycline | Antibiotic target protection |
| 3003301 | Staphylococcus aureus gyrB conferring resistance to aminocoumarin | Aminocoumarin | Cellular protection |
| 3003302 | Bartonella bacilliformis gyrB conferring resistance to aminocoumarin | Aminocoumarin | Cellular protection |
| 3003303 | Escherichia coli gyrB conferring resistance to aminocoumarin | Aminocoumarin | Cellular protection |
| 3003314 | Staphylococcus aureus parE conferring resistance to aminocoumarin | Aminocoumarin | Cellular protection |
| 3003318 | Streptomyces rishiriensis parY mutant conferring resistance to aminocoumarin | Aminocoumarin | Cellular protection |
| 3003395 | Mycobacterium tuberculosis rpsL mutations conferring resistance to Streptomycin | Aminoglycoside | Cellular protection |
| 3003445 | Mycobacterium tuberculosis tlyA mutations conferring resistance to aminoglycosides | Aminoglycoside | Cellular protection |
| 3003577 | PmrE | Aminoglycoside | Cellular protection |
| 3003578 | PmrF | Aminoglycoside | Cellular protection |
| 3003582 | PmrA | Aminoglycoside | Cellular protection |
| 3003041 | StrepSOCoccus pneumoniae PBP1a conferring resistance to amoxicillin | Beta-lactam | Cellular protection |
| 3003074 | Staphylococcus aureus cls conferring resistance to daptomycin | Beta-lactam | Cellular protection |
| 3003092 | Enterococcus faecium cls conferring resistance to daptomycin | Beta-lactam | Cellular protection |
| 3003291 | Staphylococcus aureus rpoC conferring resistance to daptomycin | Beta-lactam | Cellular protection |
| 3003357 | Clostridium difficile EF-Tu mutants conferring resistance to elfamycin | Elfamycin | Cellular protection |
| 3003438 | Enterococcus faecium EF-Tu mutants conferring resistance to GE2270A | Elfamycin | Cellular protection |
| 3003361 | Planobispora rosea EF-Tu mutants conferring resistance to inhibitor GE2270A | Elfamycin | Cellular protection |
| 3003370 | Escherichia coli EF-Tu mutants conferring resistance to Enacyloxin IIa | Elfamycin | Cellular protection |
| 3003359 | Streptomyces cinnamoneus EF-Tu mutants conferring resistance to elfamycin | Elfamycin | Cellular protection |
| 3003294 | Escherichia coli gyrA conferring resistance to Fluoroquinolones | Fluoroquinolone | Cellular protection |
| 3003295 | Mycobacterium tuberculosis gyrA conferring resistance to Fluoroquinolones | Fluoroquinolone | Cellular protection |
| 3003296 | Staphylococcus aureus gyrA conferring resistance to Fluoroquinolones | Fluoroquinolone | Cellular protection |
| 3003297 | Bartonella bacilliformis gyrA conferring resistance to Fluoroquinolones | Fluoroquinolone | Cellular protection |
| 3003298 | Mycobaterium leprae gyrA conferring resistance to Fluoroquinolones | Fluoroquinolone | Cellular protection |
| 3003304 | Mycobacterium leprae gyrB conferring resistance to Fluoroquinolone | Fluoroquinolone | Cellular protection |
| 3003305 | Ureaplasma urealyticum gyrB conferring resistance to Fluoroquinolone | Fluoroquinolone | Cellular protection |
| 3003306 | Morganella morganii gyrB conferring resistance to Fluoroquinolone | Fluoroquinolone | Cellular protection |
| 3003307 | Salmonella serovars gyrB conferring resistance to Fluoroquinolone | Fluoroquinolone | Cellular protection |
| 3003315 | Staphylococcus aureus parE conferring resistance to Fluoroquinolones | Fluoroquinolone | Cellular protection |
| 3003316 | Escherichia coli parE conferring resistance to Fluoroquinolones | Fluoroquinolone | Cellular protection |
| 3003317 | Salmonella serovars parE conferring resistance to Fluoroquinolones | Fluoroquinolone | Cellular protection |
| 3003459 | Mycobacterium tuberculosis gyrB mutant conferring resistance to Fluoroquinolone | Fluoroquinolone | Cellular protection |
| 3003684 | Pseudomonas aeruginosa gyrA conferring resistance to Fluoroquinolones | Fluoroquinolone | Cellular protection |
| 3003685 | Pseudomonas aeruginosa parE conferring resistance to Fluoroquinolones | Fluoroquinolone | Cellular protection |
| 3003789 | Campylobacter jejuni gyrA conferring resistance to Fluoroquinolones | Fluoroquinolone | Cellular protection |
| 3003924 | Haemophilus parainfluenzae gyrA conferring resistance to Fluoroquinolones | Fluoroquinolone | Cellular protection |
| 3003925 | Haemophilus parainfluenzae parC conferring resistance to Fluoroquinolones | Fluoroquinolone | Cellular protection |
| 3003926 | Salmonella enterica gyrA conferring resistance to Fluoroquinolones | Fluoroquinolone | Cellular protection |
| 3003928 | Neisseria gonorrhoeae gyrA conferring resistance to Fluoroquinolones | Fluoroquinolone | Cellular protection |
| 3003929 | Neisseria gonorrhoeae parC conferring resistance to Fluoroquinolone | Fluoroquinolone | Cellular protection |
| 3003931 | Capnocytophaga gingivalis gyrA conferring resistance to Fluoroquinolones | Fluoroquinolone | Cellular protection |
| 3003937 | Neisseria meningititis PBP2 conferring resistance to beta-lactam | Fluoroquinolone | Cellular protection |
| 3003939 | Salmonella enterica parC conferring resistance to Fluoroquinolones | Fluoroquinolone | Cellular protection |
| 3003940 | Shigella flexneri gyrA conferring resistance to Fluoroquinolones | Fluoroquinolone | Cellular protection |
| 3003941 | Shigella flexneri parC conferring resistance to Fluoroquinolones | Fluoroquinolone | Cellular protection |
| 3003974 | Propionibacterium acnes gyrA conferring resistance to Fluoroquinolones | Fluoroquinolone | Cellular protection |
| 3003970 | D-Ala-D-Ala ligase | Glycopeptide | Cellular protection |
| 3000005 | vanD | Glycopeptide | Cellular protection |
| 3000010 | vanA | Glycopeptide | Cellular protection |
| 3000013 | vanB | Glycopeptide | Cellular protection |
| 3000368 | vanC | Glycopeptide | Cellular protection |
| 3000372 | vanT | Glycopeptide | Cellular protection |
| 3002907 | vanE | Glycopeptide | Cellular protection |
| 3002909 | vanG | Glycopeptide | Cellular protection |
| 3002913 | vanO | Glycopeptide | Cellular protection |
| 3002922 | vanRC | Glycopeptide | Cellular protection |
| 3002924 | vanRE | Glycopeptide | Cellular protection |
| 3002925 | vanRF | Glycopeptide | Cellular protection |
| 3002926 | vanRG | Glycopeptide | Cellular protection |
| 3002927 | vanRL | Glycopeptide | Cellular protection |
| 3002928 | vanRM | Glycopeptide | Cellular protection |
| 3002929 | vanRN | Glycopeptide | Cellular protection |
| 3002930 | vanRO | Glycopeptide | Cellular protection |
| 3002941 | vanSO | Glycopeptide | Cellular protection |
| 3002942 | vanHA | Glycopeptide | Cellular protection |
| 3002943 | vanHB | Glycopeptide | Cellular protection |
| 3002944 | vanHD | Glycopeptide | Cellular protection |
| 3002945 | vanHF | Glycopeptide | Cellular protection |
| 3002947 | vanHM | Glycopeptide | Cellular protection |
| 3002948 | vanHO | Glycopeptide | Cellular protection |
| 3002953 | vanXM | Glycopeptide | Cellular protection |
| 3002972 | vanTG | Glycopeptide | Cellular protection |
| 3003727 | vanKI | Glycopeptide | Cellular protection |
| 3003728 | vanRI | Glycopeptide | Cellular protection |
| 3003393 | Mycobacterium tuberculosis inhA mutations conferring resistance to isoniazid | Other | Cellular protection |
| 3003889 | Escherichia coli mutant GlpT conferring resistance to fosfomycin | Other | Cellular protection |
| 3003458 | mycobacterium tuberculosis ethA mutant conferring resistance to ethionamide | Other | Cellular protection |
| 3003461 | Mycobacterium tuberculosis ndh mutant conferring resistance to isoniazid | Other | Cellular protection |
| 3003463 | Mycobacterium tuberculosis kasA mutant conferring resistance to isoniazid | Other | Cellular protection |
| 3003283 | Mycobacterium tuberculosis rpoB mutants conferring resistance to rifampicin | Rifampin | Cellular protection |
| 3003284 | Mycobacterium leprae rpoB mutants conferring resistance to rifampicin | Rifampin | Cellular protection |
| 3003285 | Staphylococcus aureus rpoB mutants conferring resistance to rifampicin | Rifampin | Cellular protection |
| 3003288 | Escherichia coli rpoB mutants conferring resistance to rifampicin | Rifampin | Cellular protection |
| 3003465 | Mycobacterium tuberculosis embB mutants conferring resistance to rifampicin | Rifampin | Cellular protection |
| 3003386 | Escherichia coli sulfonamide resistant mutant folP | Sulfonamide | Cellular protection |
| 3003387 | Streptococcus pyogenes sulfonamide resistant folP mutants | Sulfonamide | Cellular protection |
| 3003389 | Mycobacterium leprae dapsone resistant folP mutants | Sulfonamide | Cellular protection |
| 3002870 | tet34 | Tetracycline | Cellular protection |
| 3002832 | vgaD | Beta-lactam | Efflux pump |
| 3002833 | vgaE | Beta-lactam | Efflux pump |
| 3001313 | facT | Elfamycin | Efflux pump |
| 3000448 | qepA | Fluoroquinolone | Efflux pump |
| 3000600 | Erm(34) | MLSB | Efflux pump |
| 3000027 | emrA | MLSB | Efflux pump |
| 3000074 | emrB | MLSB | Efflux pump |
| 3000254 | emrY | MLSB | Efflux pump |
| 3000309 | emrD | MLSB | Efflux pump |
| 3003748 | oleC | MLSB | Efflux pump |
| 3001297 | oleR | MLSB | Efflux pump |
| 3003036 | oleB | MLSB | Efflux pump |
| 3003107 | mefB | MLSB | Efflux pump |
| 3003066 | smeR | Multidrug | Efflux pump |
| 3003067 | smeS | Multidrug | Efflux pump |
| 3000118 | vgaB | Multidrug | Efflux pump |
| 3000207 | acrA | Multidrug | Efflux pump |
| 3000491 | acrD | Multidrug | Efflux pump |
| 3000499 | acrE | Multidrug | Efflux pump |
| 3000502 | acrF | Multidrug | Efflux pump |
| 3000378 | mexB | Multidrug | Efflux pump |
| 3000776 | Enterococcus faecium adeC | Multidrug | Efflux pump |
| 3001330 | mdtD | Multidrug | Efflux pump |
| 3000777 | adeF | Multidrug | Efflux pump |
| 3000778 | adeG | Multidrug | Efflux pump |
| 3000779 | adeH | Multidrug | Efflux pump |
| 3000533 | macA | Multidrug | Efflux pump |
| 3000792 | mdtA | Multidrug | Efflux pump |
| 3000793 | mdtB | Multidrug | Efflux pump |
| 3000800 | mexC | Multidrug | Efflux pump |
| 3000801 | mexD | Multidrug | Efflux pump |
| 3000803 | mexE | Multidrug | Efflux pump |
| 3000807 | mexH | Multidrug | Efflux pump |
| 3000828 | baeR | Multidrug | Efflux pump |
| 3000829 | baeS | Multidrug | Efflux pump |
| 3000808 | mexI | Multidrug | Efflux pump |
| 3003030 | mexV | Multidrug | Efflux pump |
| 3003692 | mexJ | Multidrug | Efflux pump |
| 3003693 | mexK | Multidrug | Efflux pump |
| 3003698 | mexP | Multidrug | Efflux pump |
| 3003699 | mexQ | Multidrug | Efflux pump |
| 3003704 | mexM | Multidrug | Efflux pump |
| 3003705 | mexN | Multidrug | Efflux pump |
| 3003031 | mexW | Multidrug | Efflux pump |
| 3003033 | mexY | Multidrug | Efflux pump |
| 3003922 | oqxA | Multidrug | Efflux pump |
| 3003923 | oqxB | Multidrug | Efflux pump |
| 3000813 | mexS | Multidrug | Efflux pump |
| 3003944 | drrA | Multidrug | Efflux pump |
| 3000814 | mexT | Multidrug | Efflux pump |
| 3000804 | mexF | Multidrug | Efflux pump |
| 3000794 | mdtC | Multidrug | Efflux pump |
| 3000784 | cmeB | Multidrug | Efflux pump |
| 3000535 | macB | Multidrug | Efflux pump |
| 3000216 | acrB | Multidrug | Efflux pump |
| 3000165 | tetA | Tetracycline | Efflux pump |
| 3000561 | tet30 | Tetracycline | Efflux pump |
| 3000566 | tet39 | Tetracycline | Efflux pump |
| 3000569 | tet41 | Tetracycline | Efflux pump |
| 3000573 | tet43 | Tetracycline | Efflux pump |
| 3000481 | tet35 | Tetracycline | Efflux pump |
| 3000173 | tetE | Tetracycline | Efflux pump |
| 3000180 | tetA(P) | Tetracycline | Efflux pump |
| 3000181 | tetV | Tetracycline | Efflux pump |
| 3003830 | aminocoumarin resistant alaS | Aminocoumarin | Other |
| 3003840 | aminocoumarin resistant cysB | Aminocoumarin | Other |
| 3001293 | desR | Other | Other |

**Table S2** Soil properties and microbial biomass among distinct crop rotational regimes and complexity (n = 3); values are mean ± standard deviation.

| Soil properties | A4PoW3 | BL | CWWM | PWWM | W | Regimes *P* | | Regimes F | Complexity *P* | Complexity F |
| --- | --- | --- | --- | --- | --- | --- | --- | --- | --- | --- |
| SWC | 17.52 ± 0.29 ab | 18.48 ± 0.34 a | 18.3 ± 0.05 ab | 17.29 ± 0.54 b | 18.3 ± 0.28 ab | 0.11 | 2.53 | | 0.09 | 3.12 |
| pH | 8.46 ± 0.01 c | 8.55 ± 0 a | 8.43 ± 0.01 d | 8.46 ± 0.01 c | 8.51 ± 0.01 b | **<0.01** | 54.68 | | **<0.01** | 119.79 |
| SOC(g/kg) | 11 ± 0.12 a | 7.61 ± 0.05 c | 11.18 ± 0.99 a | 9.06 ± 0.22 b | 9.51 ± 0.14 b | **<0.01** | 10.27 | | **<0.01** | 11.10 |
| C/N | 15.5 ± 0.11 d | 19.49 ± 0.05 a | 15.85 ± 0.19 d | 17.19 ± 0.12 c | 18.07 ± 0.29 b | **<0.01** | 88.60 | | **<0.01** | 146.51 |
| PCM/PNM | 5.46 ± 0.21 b | 5.79 ± 0.53 b | 9.95 ± 0.68 a | 5.82 ± 0.34 b | 4.54 ± 0.31 b | **<0.01** | 22.25 | | **<0.01** | 25.48 |
| PON(g/kg) | 0.43 ± 0.03 ab | 0.17 ± 0.01 c | 0.54 ± 0.08 a | 0.29 ± 0.04 bc | 0.21 ± 0.03 c | **<0.01** | 11.22 | | **<0.01** | 13.82 |
| NH_4_^+^-N（mg/kg） | 16.8 ± 1.18 a | 18.25 ± 1 a | 17.19 ± 1.23 a | 20.23 ± 1.76 a | 19.1 ± 1.52 a | 0.43 | 1.05 | | 0.36 | 1.22 |
| NO_3_^-^-N(mg/kg) | 9.95 ± 0.57 a | 10.15 ± 0.63 a | 12.5 ± 2.6 a | 8.9 ± 0.11 a | 8.83 ± 2.19 a | 0.50 | 0.90 | | 0.41 | 1.07 |
| MBC(mg/kg) | 351.17 ± 2.44 ab | 184.91 ± 11.62 b | 299.95 ± 12.91 ab | 438.98 ± 145.51 a | 248.65 ± 15.19 ab | 0.15 | 2.17 | | 0.06 | 3.62 |
| MBN(mg/kg) | 57.67 ± 1.45 b | 41.4 ± 1.94 c | 63.83 ± 1.19 a | 58.7 ± 0.63 b | 39.83 ± 2.21 c | **<0.01** | 47.36 | | **<0.01** | 47.83 |

Note: A4PoW3, alfalfa-potato-winter wheat. BL, bare land; CWWM, corn-winter wheat-winter wheat-millet; PWWM, pea-winter wheat-winter wheat-millet; W, continuous winter wheat; SWC, soil water content; pH, soil pH; SOC, total organic carbon; C/N, total carbon/nitrogen ratio; PCM/PNM, potential carbon mineralization/potential nitrogen mineralization ratio; PON, particulate organic nitrogen; NH_4_^+^-N, ammonium nitrogen; NO_3_^-^-N, nitrate nitrogen; MBC, soil microbial biomass carbon; MBN, soil microbial biomass nitrogen. Regimes, five crop rotational regimes; Complexity, four levels of crop rotation. Values within the same column followed by different letters indicate significant differences. Bold *P-values* indicate significant differences (*P*< 0.05; ANOVA, Tukey’s HSD test)

**Table S3** The abundance of antibiotic resistance genes among distinct crop rotational regimes (n = 3). Values are mean ± standard deviation.

| **ARG type** | **ARG subtype** | **A4PoW3** | **BL** | **CWWM** | **PWWM** | **W** | ***P*** | **F** |
| --- | --- | --- | --- | --- | --- | --- | --- | --- |
| Aminocoumarin | aminocoumarin resistant alaS | 5928 ± 190.08 a | 5258.67 ± 214.65 a | 5838.67 ± 8.67 a | 5896.67 ± 113.24 a | 5696 ± 178.26 a | 0.15 | 1.88 |
|  | aminocoumarin resistant cysB | 176 ± 11.72 ab | 140.67 ± 22.04 ab | 185.33 ± 28.39 ab | 172 ± 11.37 ab | 122.67 ± 8.67 b | 0.25 | 1.51 |
|  | Staphylococcus aureus gyrB conferring resistance to aminocoumarin | 1705.33 ± 84.42 a | 1495.33 ± 11.1 abc | 1659.33 ± 28.15 ab | 1682.67 ± 27.28 ab | 1586.67 ± 106.23 abc | 0.09 | 2.38 |
|  | Bartonella bacilliformis gyrB conferring resistance to aminocoumarin | 1098.67 ± 21.05 a | 930 ± 36.9 b | 1164 ± 68.07 a | 1072.67 ± 42.53 a | 1167.33 ± 41.33 a | **0.01** | 4.26 |
|  | Escherichia coli gyrB conferring resistance to aminocoumarin | 816 ± 31.64 a | 612.67 ± 33.91 b | 804 ± 34.02 a | 687.33 ± 42.81 b | 646.67 ± 61.37 b | **0.01** | 4.23 |
|  | Staphylococcus aureus parE conferring resistance to aminocoumarin | 190 ± 11.14 a | 160 ± 8.08 a | 177.33 ± 4.06 a | 178.67 ± 1.33 a | 168.67 ± 17.33 a | 0.46 | 1.01 |
|  | Streptomyces rishiriensis parY mutant conferring resistance to aminocoumarin | 365.33 ± 23.56 a | 288.67 ± 18.67 b | 398.67 ± 21.18 a | 397.33 ± 13.78 a | 407.33 ± 31.21 a | **0.03** | 3.50 |
| Aminoglycoside | AAC(6')-Iaj | 8 ± 3.06 c | 16 ± 3.06 ab | 18 ± 2 ab | 22.67 ± 0.67 a | 14.67 ± 1.76 bc | **0.02** | 3.60 |
|  | AAC(2')-Ib | 69.33 ± 12.35 a | 55.33 ± 2.4 a | 78.67 ± 5.46 a | 81.33 ± 5.33 a | 88 ± 5.03 a | 0.40 | 1.12 |
|  | AAC(2')-Ic | 34.67 ± 2.4 ab | 23.33 ± 4.37 b | 40.67 ± 4.81 ab | 34 ± 3.46 ab | 28 ± 4 ab | 0.44 | 1.05 |
|  | AAC(3)-Ic | 33.33 ± 2.91 bc | 24.67 ± 3.53 c | 42.67 ± 5.46 ab | 32.67 ± 4.67 bc | 51.33 ± 9.68 a | **0.03** | 3.36 |
|  | AAC(3)-IV | 78 ± 13.11 ab | 66 ± 1.15 b | 94 ± 9.02 a | 74.67 ± 5.46 ab | 68.67 ± 6.57 ab | 0.09 | 2.36 |
|  | AAC(3)-VIIa | 36 ± 3.06 a | 48 ± 8.08 a | 39.33 ± 2.91 a | 50 ± 8.72 a | 38.67 ± 4.06 a | 0.54 | 0.87 |
|  | AAC(3)-VIIIa | 48.67 ± 5.81 a | 36.67 ± 6.57 a | 50 ± 13.32 a | 34 ± 3.06 a | 49.33 ± 14.34 a | 0.74 | 0.58 |
|  | AAC(6')-Ib-SK | 255.33 ± 21.83 a | 204.67 ± 9.82 ab | 207.33 ± 0.67 ab | 236 ± 4.16 ab | 196 ± 22.74 ab | 0.20 | 1.66 |
|  | ANT(9)-Ia | 67.33 ± 7.42 a | 38 ± 4.16 c | 41.33 ± 2.91 bc | 51.33 ± 9.82 abc | 55.33 ± 4.67 ab | **0.03** | 3.45 |
|  | APH(6)-Ia | 69.33 ± 8.67 a | 62 ± 8.72 a | 66 ± 13.01 a | 67.33 ± 3.53 a | 70 ± 6.11 a | 0.82 | 0.47 |
|  | VgbB | 55.33 ± 4.67 a | 36 ± 9.87 a | 40 ± 7.21 a | 42 ± 7.02 a | 44 ± 0 a | 0.61 | 0.77 |
|  | vatF | 68.67 ± 10.73 ab | 51.33 ± 10.97 b | 74.67 ± 7.06 ab | 89.33 ± 16.83 a | 62 ± 3.06 ab | 0.32 | 1.29 |
|  | APH(9)-Ia | 41.33 ± 7.42 a | 49.33 ± 9.33 a | 54 ± 5.77 a | 57.33 ± 4.67 a | 49.33 ± 4.67 a | 0.85 | 0.42 |
|  | APH(6)-Ic | 62 ± 3.06 a | 56 ± 17.09 a | 54 ± 10.26 a | 74 ± 8 a | 55.33 ± 2.4 a | 0.74 | 0.58 |
|  | AAC(3)-Xa | 42.67 ± 5.93 a | 40.67 ± 10.35 a | 43.33 ± 8.35 a | 50 ± 7.57 a | 54 ± 11.72 a | 0.86 | 0.42 |
|  | AAC(6')-If | 19.33 ± 2.4 a | 14 ± 0 a | 19.33 ± 9.4 a | 17.33 ± 1.76 a | 9.33 ± 5.46 a | 0.40 | 1.12 |
|  | AAC(6')-Ii | 19.33 ± 4.37 a | 26.67 ± 8.74 a | 29.33 ± 2.91 a | 28.67 ± 8.67 a | 26 ± 5.03 a | 0.77 | 0.54 |
|  | AAC(6')-Isa | 130 ± 11.55 a | 84 ± 7.21 a | 118.67 ± 17.33 a | 110.67 ± 15.38 a | 112 ± 5.03 a | 0.26 | 1.46 |
|  | Mycobacterium tuberculosis rpsL mutations conferring resistance to Streptomycin | 1082 ± 71.84 a | 942.67 ± 62.63 a | 1086 ± 15.87 a | 1064.67 ± 18.41 a | 990 ± 45.18 a | 0.42 | 1.08 |
|  | Mycobacterium tuberculosis tlyA mutations conferring resistance to aminoglycosides | 1417.33 ± 61.54 a | 1208.67 ± 35.89 a | 1296.67 ± 18.49 a | 1354.67 ± 96.34 a | 1332.67 ± 27.55 a | 0.50 | 0.93 |
|  | PmrE | 512 ± 22.27 a | 438 ± 61.61 a | 464.67 ± 10.73 a | 477.33 ± 24.5 a | 408.67 ± 35.35 a | 0.45 | 1.01 |
|  | PmrF | 800 ± 59.41 a | 633.33 ± 25.96 bc | 708 ± 7.57 ab | 680 ± 38.57 ab | 657.33 ± 47.35 bc | **0.02** | 3.88 |
|  | PmrA | 2111.33 ± 158.91 a | 1600 ± 83.53 b | 1900 ± 73.93 ab | 1944.67 ± 70.62 ab | 1788.67 ± 63.61 ab | 0.17 | 1.82 |
| Beta-lactam | FEZ-1 beta-lactamase | 52.67 ± 3.71 ab | 43.33 ± 10.48 b | 60.67 ± 13.78 ab | 68.67 ± 7.42 ab | 74.67 ± 13.68 a | 0.20 | 1.69 |
|  | BJP-1 | 50 ± 7.57 ab | 38 ± 5.29 b | 52.67 ± 5.33 ab | 47.33 ± 7.42 ab | 66 ± 11.14 a | 0.07 | 2.55 |
|  | LRA-1 | 18.67 ± 7.86 a | 3.33 ± 1.76 b | 12.67 ± 2.4 ab | 10 ± 1.15 ab | 8.67 ± 2.67 ab | 0.35 | 1.23 |
|  | LRA-5 | 10.67 ± 5.21 a | 9.33 ± 3.53 a | 8 ± 1.15 a | 10.67 ± 4.81 a | 13.33 ± 4.37 a | 0.90 | 0.35 |
|  | LRA-17 | 10.67 ± 1.33 ab | 5.33 ± 2.4 b | 20 ± 9.24 a | 12.67 ± 3.33 ab | 7.33 ± 1.33 ab | 0.23 | 1.56 |
|  | LRA-19 | 7.33 ± 1.76 ab | 10.67 ± 2.91 ab | 14 ± 4.62 ab | 10 ± 3.46 ab | 6 ± 2.31 b | 0.29 | 1.37 |
|  | NmcR | 158 ± 8.33 a | 176 ± 24.44 a | 189.33 ± 13.78 a | 182.67 ± 4.67 a | 168.67 ± 11.22 a | 0.36 | 1.21 |
|  | SPG-1 | 18.67 ± 9.68 a | 18 ± 6.43 a | 11.33 ± 5.46 a | 16 ± 3.06 a | 14 ± 2.31 a | 0.89 | 0.36 |
|  | Rm3 beta-lactamase | 13.33 ± 4.37 a | 14.67 ± 5.7 a | 18 ± 5.29 a | 18 ± 2.31 a | 17.33 ± 6.57 a | 0.91 | 0.34 |
|  | Streptococcus pneumoniae PBP1a conferring resistance to amoxicillin | 1885.33 ± 116.15 a | 1654 ± 53.3 a | 1794.67 ± 52.41 a | 1868.67 ± 29.24 a | 1750 ± 50.33 a | 0.38 | 1.17 |
|  | mecB | 91.33 ± 11.1 a | 100.67 ± 9.61 a | 108 ± 6.11 a | 111.33 ± 9.82 a | 98 ± 26.23 a | 0.93 | 0.29 |
|  | mecA | 98.67 ± 12.13 a | 74.67 ± 3.71 a | 92.67 ± 8.67 a | 80 ± 8.33 a | 84.67 ± 2.4 a | 0.53 | 0.89 |
|  | Staphylococcus aureus cls conferring resistance to daptomycin | 573.33 ± 19.37 ab | 500.67 ± 76.91 bc | 637.33 ± 53.28 a | 554 ± 13.32 abc | 452.67 ± 33.95 c | 0.11 | 2.19 |
|  | Enterococcus faecium cls conferring resistance to daptomycin | 76.67 ± 4.06 a | 69.33 ± 8.67 a | 78 ± 7.02 a | 85.33 ± 4.37 a | 83.33 ± 8.97 a | 0.54 | 0.87 |
|  | Staphylococcus aureus rpoC conferring resistance to daptomycin | 11291.33 ± 500.26 a | 9510.67 ± 369.59 c | 10832 ± 82.95 ab | 10427.33 ± 127.94 abc | 10420 ± 321.75 abc | 0.07 | 2.51 |
|  | vgaD | 115.33 ± 5.81 bcd | 123.33 ± 9.33 bc | 124.67 ± 2.91 bc | 147.33 ± 2.4 a | 136 ± 2 ab | **0.01** | 4.75 |
|  | vgaE | 415.33 ± 24.39 a | 303.33 ± 26.34 b | 413.33 ± 30.38 a | 332.67 ± 21.98 ab | 342.67 ± 22.64 ab | 0.28 | 1.40 |
| Elfamycin | Clostridium difficile EF-Tu mutants conferring resistance to elfamycin | 303.33 ± 3.71 a | 234.67 ± 6.57 bc | 265.33 ± 27.38 ab | 219.33 ± 6.67 c | 240.67 ± 16.83 bc | **0.02** | 3.69 |
|  | Enterococcus faecium EF-Tu mutants conferring resistance to GE2270A | 2303.33 ± 146.09 a | 2251.33 ± 82.9 a | 2305.33 ± 58.77 a | 2182.67 ± 50.34 a | 2242.67 ± 106.82 a | 0.65 | 0.71 |
|  | Planobispora rosea EF-Tu mutants conferring resistance to inhibitor GE2270A | 453.33 ± 19.37 ab | 392.67 ± 15.38 b | 437.33 ± 14.25 ab | 466 ± 44.74 ab | 490 ± 26.63 a | 0.20 | 1.68 |
|  | Escherichia coli EF-Tu mutants conferring resistance to Enacyloxin IIa | 2712 ± 97.08 a | 2388 ± 117.05 b | 2695.33 ± 61.2 a | 2561.33 ± 23.22 ab | 2502 ± 177.99 ab | 0.29 | 1.37 |
|  | Streptomyces cinnamoneus EF-Tu mutants conferring resistance to elfamycin | 358.67 ± 27.84 bc | 372.67 ± 8.67 abc | 360 ± 11.02 abc | 382 ± 6.43 abc | 423.33 ± 8.74 a | 0.12 | 2.12 |
|  | facT | 636 ± 82.03 a | 460 ± 47.72 a | 571.33 ± 49.27 a | 603.33 ± 20.8 a | 594 ± 24 a | 0.46 | 1.00 |
| Fluoroquinolone | Escherichia coli gyrA conferring resistance to Fluoroquinolones | 48 ± 1.15 ab | 35.33 ± 6.36 b | 54 ± 6.43 a | 51.33 ± 8.67 ab | 54 ± 6 a | 0.37 | 1.17 |
|  | Mycobacterium tuberculosis gyrA conferring resistance to Fluoroquinolones | 1226 ± 43.1 a | 997.33 ± 38.75 a | 1056 ± 63.79 a | 1098 ± 25.4 a | 1048 ± 50.48 a | 0.45 | 1.02 |
|  | Staphylococcus aureus gyrA conferring resistance to Fluoroquinolones | 1587.33 ± 52.53 a | 1384.67 ± 13.48 ab | 1547.33 ± 33.91 ab | 1516 ± 52.42 ab | 1502.67 ± 54.41 ab | 0.16 | 1.87 |
|  | Bartonella bacilliformis gyrA conferring resistance to Fluoroquinolones | 2254 ± 50.71 a | 1938 ± 98.85 b | 2122 ± 17.32 ab | 2194.67 ± 7.42 a | 2094.67 ± 100.81 ab | 0.19 | 1.73 |
|  | Mycobaterium leprae gyrA conferring resistance to Fluoroquinolones | 522.67 ± 47.75 a | 470.67 ± 20.73 a | 475.33 ± 14.11 a | 522 ± 34.87 a | 423.33 ± 46.34 a | 0.59 | 0.79 |
|  | Mycobacterium leprae gyrB conferring resistance to Fluoroquinolone | 480 ± 16.65 a | 374.67 ± 32.69 b | 440 ± 24.44 ab | 432.67 ± 7.42 ab | 447.33 ± 14.62 ab | 0.08 | 2.38 |
|  | Ureaplasma urealyticum gyrB conferring resistance to Fluoroquinolone | 260 ± 8.33 a | 218.67 ± 27.48 ab | 246.67 ± 14.62 a | 253.33 ± 9.4 a | 217.33 ± 13.68 ab | 0.09 | 2.30 |
|  | Morganella morganii gyrB conferring resistance to Fluoroquinolone | 934.67 ± 74.75 a | 782 ± 67.3 b | 852.67 ± 36.04 ab | 739.33 ± 11.85 b | 726 ± 16 b | 0.08 | 2.40 |
|  | Salmonella serovars gyrB conferring resistance to Fluoroquinolone | 1420.67 ± 50.99 a | 1104 ± 30.35 c | 1298.67 ± 30.64 ab | 1268 ± 39.11 abc | 1216.67 ± 64.4 bc | **0.03** | 3.34 |
|  | Staphylococcus aureus parE conferring resistance to Fluoroquinolones | 14 ± 8.08 a | 8 ± 1.15 a | 6.67 ± 3.71 a | 7.33 ± 3.71 a | 9.33 ± 4.67 a | 0.75 | 0.56 |
|  | Escherichia coli parE conferring resistance to Fluoroquinolones | 76.67 ± 12.77 a | 66.67 ± 10.09 a | 77.33 ± 13.38 a | 74 ± 2 a | 58.67 ± 6.96 a | 0.49 | 0.95 |
|  | Salmonella serovars parE conferring resistance to Fluoroquinolones | 166 ± 8.08 a | 147.33 ± 18.56 a | 148 ± 11.72 a | 152 ± 15.87 a | 139.33 ± 9.4 a | 0.85 | 0.42 |
|  | Mycobacterium tuberculosis gyrB mutant conferring resistance to Fluoroquinolone | 1155.33 ± 53.53 a | 940.67 ± 64.06 a | 1118.67 ± 5.81 a | 1143.33 ± 31.52 a | 1096.67 ± 68.21 a | 0.36 | 1.20 |
|  | Pseudomonas aeruginosa gyrA conferring resistance to Fluoroquinolones | 1110 ± 30.35 a | 916 ± 37.75 b | 974 ± 3.06 b | 1011.33 ± 38.39 ab | 922 ± 43 b | 0.06 | 2.69 |
|  | Pseudomonas aeruginosa parE conferring resistance to Fluoroquinolones | 462.67 ± 16.9 a | 351.33 ± 29.54 b | 420 ± 38.94 ab | 400 ± 8.72 ab | 385.33 ± 17.33 ab | 0.25 | 1.51 |
|  | Campylobacter jejuni gyrA conferring resistance to Fluoroquinolones | 232 ± 29.48 a | 237.33 ± 22.52 a | 248.67 ± 15.03 a | 210.67 ± 10.09 a | 266 ± 9.87 a | 0.57 | 0.83 |
|  | Haemophilus parainfluenzae gyrA conferring resistance to Fluoroquinolones | 162 ± 8.33 a | 152.67 ± 6.96 a | 168 ± 9.87 a | 153.33 ± 19.4 a | 158 ± 19.08 a | 0.73 | 0.60 |
|  | Haemophilus parainfluenzae parC conferring resistance to Fluoroquinolones | 50.67 ± 4.37 ab | 38 ± 5.29 b | 36.67 ± 9.33 b | 58 ± 18.15 ab | 35.33 ± 5.81 b | 0.08 | 2.39 |
|  | Salmonella enterica gyrA conferring resistance to Fluoroquinolones | 847.33 ± 22.52 a | 651.33 ± 82.9 b | 807.33 ± 53.18 a | 778 ± 46.13 ab | 702 ± 63.79 ab | 0.16 | 1.84 |
|  | Neisseria gonorrhoeae gyrA conferring resistance to Fluoroquinolones | 192 ± 8.08 a | 172 ± 18.15 ab | 196.67 ± 17.02 a | 188.67 ± 4.67 ab | 142 ± 8.08 b | 0.12 | 2.10 |
|  | Neisseria gonorrhoeae parC conferring resistance to Fluoroquinolone | 294.67 ± 34.72 ab | 237.33 ± 21.36 ab | 300.67 ± 36.68 ab | 260 ± 8.08 ab | 225.33 ± 12.13 b | 0.27 | 1.44 |
|  | Capnocytophaga gingivalis gyrA conferring resistance to Fluoroquinolones | 247.33 ± 17.9 a | 160 ± 14.74 c | 223.33 ± 16.18 ab | 231.33 ± 18.12 ab | 222 ± 17.01 ab | **0.03** | 3.31 |
|  | Neisseria meningititis PBP2 conferring resistance to beta-lactam | 1378 ± 65.43 a | 1118.67 ± 109.14 b | 1266.67 ± 45.35 ab | 1268.67 ± 19.06 ab | 1178 ± 27.74 ab | 0.34 | 1.24 |
|  | Salmonella enterica parC conferring resistance to Fluoroquinolones | 126.67 ± 22.78 a | 98.67 ± 13.13 ab | 94 ± 12.86 ab | 97.33 ± 1.76 ab | 84.67 ± 3.71 b | 0.34 | 1.26 |
|  | Shigella flexneri gyrA conferring resistance to Fluoroquinolones | 109.33 ± 4.67 a | 90 ± 10.39 a | 98 ± 14.19 a | 100 ± 2.31 a | 108.67 ± 5.81 a | 0.64 | 0.72 |
|  | Shigella flexneri parC conferring resistance to Fluoroquinolones | 41.33 ± 7.51 a | 26 ± 5.29 a | 32.67 ± 7.06 a | 26 ± 5.29 a | 40.67 ± 6.36 a | 0.40 | 1.13 |
|  | Propionibacterium acnes gyrA conferring resistance to Fluoroquinolones | 462 ± 35.55 abc | 377.33 ± 23.7 c | 414 ± 16.17 bc | 534.67 ± 21.67 a | 484.67 ± 12.13 ab | **0.05** | 2.80 |
|  | qepA | 1569.33 ± 144.29 a | 1320.67 ± 63.67 a | 1456.67 ± 79.54 a | 1475.33 ± 55.53 a | 1337.33 ± 40.76 a | 0.34 | 1.24 |
| Glycopeptide | D-Ala-D-Ala ligase | 1343.33 ± 66.36 a | 1075.33 ± 76.35 a | 1282.67 ± 46.77 a | 1214.67 ± 45.67 a | 1205.33 ± 85.68 a | 0.33 | 1.28 |
|  | vanD | 131.33 ± 20.8 ab | 144.67 ± 1.76 ab | 126.67 ± 7.69 ab | 173.33 ± 16.83 a | 115.33 ± 10.35 b | 0.27 | 1.44 |
|  | vanA | 57.33 ± 1.76 a | 50.67 ± 4.37 a | 52.67 ± 4.67 a | 68 ± 3.46 a | 60 ± 9.17 a | 0.45 | 1.02 |
|  | vanB | 6 ± 0 ab | 13.33 ± 4.67 a | 7.33 ± 1.76 ab | 8 ± 3.06 ab | 12.67 ± 2.4 a | 0.10 | 2.22 |
|  | vanC | 22 ± 10.26 a | 38.67 ± 7.86 a | 25.33 ± 6.57 a | 23.33 ± 5.46 a | 19.33 ± 4.81 a | 0.62 | 0.75 |
|  | vanT | 23.33 ± 1.76 b | 48 ± 6.11 a | 26.67 ± 4.37 b | 28 ± 1.15 b | 32 ± 2.31 b | **0.03** | 3.45 |
|  | vanE | 23.33 ± 1.33 a | 14.67 ± 0.67 a | 22 ± 6.43 a | 17.33 ± 2.91 a | 17.33 ± 2.67 a | 0.66 | 0.70 |
|  | vanG | 124 ± 8.08 abc | 162 ± 10.07 a | 120 ± 17.78 bc | 123.33 ± 12.72 bc | 157.33 ± 5.46 ab | 0.12 | 2.12 |
|  | vanO | 112.67 ± 12.98 a | 83.33 ± 9.33 a | 111.33 ± 9.68 a | 113.33 ± 15.68 a | 108 ± 7.57 a | 0.26 | 1.47 |
|  | vanRC | 24 ± 2 a | 15.33 ± 4.67 a | 16.67 ± 4.81 a | 25.33 ± 6.77 a | 22.67 ± 6.57 a | 0.59 | 0.79 |
|  | vanRE | 32.67 ± 4.81 a | 20.67 ± 7.69 ab | 23.33 ± 4.67 ab | 26.67 ± 3.53 ab | 25.33 ± 5.81 ab | 0.48 | 0.97 |
|  | vanRF | 152 ± 15.1 a | 150.67 ± 8.35 a | 140 ± 7.57 a | 144.67 ± 9.26 a | 130 ± 15.53 a | 0.90 | 0.34 |
|  | vanRG | 14.67 ± 2.4 a | 14.67 ± 2.4 a | 14 ± 4 a | 12.67 ± 2.4 a | 21.33 ± 4.81 a | 0.64 | 0.71 |
|  | vanRL | 16.67 ± 8.67 ab | 25.33 ± 1.76 a | 10.67 ± 0.67 b | 22.67 ± 3.33 a | 14 ± 3.06 ab | 0.06 | 2.76 |
|  | vanRM | 151.33 ± 18.27 a | 120 ± 27.59 a | 132.67 ± 12.35 a | 136.67 ± 13.87 a | 148 ± 30.09 a | 0.96 | 0.23 |
|  | vanRN | 14 ± 4.62 ab | 14 ± 5.03 ab | 12 ± 2 ab | 15.33 ± 2.4 a | 8.67 ± 1.76 ab | 0.37 | 1.19 |
|  | vanRO | 832.67 ± 45.76 a | 582 ± 61.85 c | 721.33 ± 33.19 abc | 782.67 ± 32.36 ab | 736 ± 59 abc | 0.13 | 2.01 |
|  | vanSO | 696.67 ± 39.28 a | 529.33 ± 27.28 b | 695.33 ± 16.18 a | 688 ± 27.23 a | 651.33 ± 43.36 ab | 0.18 | 1.75 |
|  | vanHA | 355.33 ± 23.13 a | 297.33 ± 35.95 a | 345.33 ± 34.74 a | 355.33 ± 11.57 a | 366.67 ± 21.49 a | 0.48 | 0.97 |
|  | vanHB | 306 ± 14.74 a | 284 ± 17.24 a | 289.33 ± 13.53 a | 294.67 ± 14.53 a | 228 ± 2 a | 0.65 | 0.71 |
|  | vanHD | 81.33 ± 4.67 ab | 90 ± 7.02 a | 93.33 ± 8.11 a | 92 ± 4.16 a | 62.67 ± 9.33 b | 0.06 | 2.70 |
|  | vanHF | 152.67 ± 8.51 a | 110 ± 10.39 b | 128 ± 17.47 ab | 130 ± 8.33 ab | 120.67 ± 9.82 ab | 0.12 | 2.06 |
|  | vanHM | 36.67 ± 6.96 a | 36 ± 6.11 a | 36.67 ± 5.7 a | 36.67 ± 10.41 a | 36 ± 6.11 a | 0.58 | 0.80 |
|  | vanHO | 88 ± 10.07 ab | 80.67 ± 5.7 b | 77.33 ± 4.67 b | 104.67 ± 4.37 a | 94 ± 3.06 ab | 0.12 | 2.07 |
|  | vanXM | 38 ± 3.06 a | 22.67 ± 1.33 a | 30.67 ± 11.68 a | 35.33 ± 4.81 a | 32.67 ± 9.61 a | 0.85 | 0.43 |
|  | vanTG | 453.33 ± 19.74 a | 314.67 ± 29.9 b | 374 ± 19.73 ab | 396 ± 29.46 ab | 333.33 ± 22.93 b | 0.17 | 1.79 |
|  | vanKI | 70.67 ± 10.73 a | 52.67 ± 10.97 a | 62 ± 10.58 a | 66.67 ± 4.67 a | 59.33 ± 3.53 a | 0.50 | 0.94 |
|  | vanRI | 298 ± 27.15 ab | 326 ± 4.16 a | 276.67 ± 11.1 ab | 282.67 ± 17.14 ab | 273.33 ± 15.38 b | 0.12 | 2.07 |
| MLSB | Erm(34) | 18.67 ± 2.67 a | 5.33 ± 0.67 b | 8.67 ± 2.91 b | 14 ± 3.06 ab | 12.67 ± 4.37 ab | **0.05** | 2.81 |
|  | emrA | 12.67 ± 0.67 a | 8.67 ± 3.53 a | 8.67 ± 2.91 a | 9.33 ± 2.91 a | 8.67 ± 2.91 a | 0.49 | 0.95 |
|  | emrB | 322.67 ± 40.5 a | 296 ± 42.72 a | 308 ± 19.01 a | 308 ± 12.06 a | 256 ± 22.54 a | 0.42 | 1.08 |
|  | emrY | 48 ± 15.62 a | 52.67 ± 11.68 a | 56.67 ± 7.86 a | 54.67 ± 7.51 a | 48.67 ± 0.67 a | 0.69 | 0.65 |
|  | emrD | 14 ± 1.15 b | 24.67 ± 2.4 a | 17.33 ± 5.46 ab | 15.33 ± 0.67 ab | 15.33 ± 5.46 ab | 0.18 | 1.75 |
|  | oleC | 7878 ± 465.86 a | 6512.67 ± 288.95 a | 7444.67 ± 307.34 a | 7798 ± 131.49 a | 7101.33 ± 195.37 a | 0.25 | 1.50 |
|  | oleR | 344 ± 11.37 ab | 228.67 ± 12.13 c | 348 ± 10.07 ab | 382.67 ± 27.24 a | 294.67 ± 17.33 bc | **0.01** | 4.18 |
|  | oleB | 609.33 ± 22.34 a | 442.67 ± 31.92 b | 610.67 ± 29.94 a | 618.67 ± 37.78 a | 592 ± 31.39 a | 0.07 | 2.57 |
|  | mefB | 16 ± 2.31 ab | 15.33 ± 3.33 ab | 21.33 ± 0.67 a | 16 ± 2 ab | 9.33 ± 3.53 b | 0.11 | 2.13 |
| Multidrug | smeR | 446 ± 26.63 a | 378.67 ± 37.88 ab | 408.67 ± 29.94 ab | 370.67 ± 23.79 ab | 361.33 ± 34.65 b | 0.36 | 1.20 |
|  | smeS | 634.67 ± 56.05 a | 508 ± 23.07 b | 546 ± 56 ab | 574.67 ± 33.65 ab | 492 ± 21.2 b | 0.19 | 1.73 |
|  | vgaB | 140 ± 3.46 a | 124 ± 14.74 a | 142.67 ± 25.73 a | 121.33 ± 9.68 a | 122 ± 11.37 a | 0.66 | 0.70 |
|  | acrA | 36.67 ± 9.26 ab | 30.67 ± 3.71 abc | 38 ± 1.15 a | 20 ± 1.15 c | 23.33 ± 1.76 bc | 0.13 | 2.05 |
|  | acrD | 60 ± 17.47 a | 24.67 ± 5.21 c | 49.33 ± 4.37 ab | 44.67 ± 1.76 abc | 37.33 ± 7.69 abc | 0.12 | 2.10 |
|  | acrE | 13.33 ± 0.67 a | 8 ± 1.15 a | 14 ± 2 a | 15.33 ± 3.33 a | 12 ± 3.06 a | 0.57 | 0.83 |
|  | acrF | 40 ± 4.62 ab | 24.67 ± 6.67 ab | 42.67 ± 8.74 ab | 43.33 ± 7.42 a | 39.33 ± 3.33 ab | 0.21 | 1.63 |
|  | mexB | 175.33 ± 18.27 a | 124 ± 8 c | 152.67 ± 11.1 abc | 146 ± 8.08 abc | 130.67 ± 12.77 bc | 0.12 | 2.07 |
|  | Enterococcus faecium adeC | 1194 ± 109.12 a | 972.67 ± 51.51 ab | 1009.33 ± 24.01 ab | 1116.67 ± 69.19 ab | 956.67 ± 41.33 ab | 0.37 | 1.17 |
|  | mdtD | 248 ± 50.29 a | 214.67 ± 18.67 a | 244.67 ± 5.81 a | 241.33 ± 17.9 a | 201.33 ± 6.96 a | 0.85 | 0.42 |
|  | adeF | 213.33 ± 20.34 ab | 166 ± 12 b | 233.33 ± 18.99 a | 180.67 ± 14.62 b | 176.67 ± 17.68 b | 0.11 | 2.19 |
|  | adeG | 682.67 ± 28.39 ab | 558.67 ± 44.2 c | 704 ± 17.32 a | 643.33 ± 31.88 abc | 610.67 ± 24.94 bc | **0.04** | 3.05 |
|  | adeH | 248.67 ± 26.39 a | 226 ± 29.05 ab | 222 ± 38.7 ab | 174 ± 18.04 bc | 150 ± 3.46 c | 0.12 | 2.12 |
|  | macA | 110 ± 16.04 a | 85.33 ± 11.62 a | 103.33 ± 14.71 a | 117.33 ± 8.19 a | 100.67 ± 16.83 a | 0.49 | 0.95 |
|  | mdtA | 248.67 ± 12.98 a | 236 ± 34.78 a | 238.67 ± 8.74 a | 234 ± 34.95 a | 196 ± 9.17 a | 0.51 | 0.91 |
|  | mdtB | 1540.67 ± 26.09 a | 1344.67 ± 80.01 b | 1538 ± 45.03 a | 1420.67 ± 39.75 ab | 1344.67 ± 83.48 b | 0.10 | 2.26 |
|  | mexC | 54 ± 8 a | 20.67 ± 2.4 c | 33.33 ± 6.96 bc | 30.67 ± 9.82 bc | 27.33 ± 1.76 bc | **0.03** | 3.30 |
|  | mexD | 92 ± 9.17 ab | 38 ± 7.02 c | 92 ± 14.05 ab | 80 ± 2.31 ab | 64 ± 8.08 bc | **0.01** | 4.92 |
|  | mexE | 236.67 ± 19.88 a | 186.67 ± 10.48 b | 207.33 ± 10.09 ab | 174.67 ± 5.93 b | 190.67 ± 6.57 b | **0.04** | 3.13 |
|  | mexH | 72.67 ± 2.4 ab | 77.33 ± 7.51 a | 65.33 ± 5.21 abc | 78.67 ± 12.02 a | 74 ± 10.07 ab | 0.06 | 2.71 |
|  | baeR | 465.33 ± 31.8 ab | 408.67 ± 14.53 bc | 481.33 ± 17.9 a | 428.67 ± 6.36 abc | 409.33 ± 12.67 bc | 0.10 | 2.25 |
|  | baeS | 816 ± 120.95 a | 743.33 ± 36.52 a | 829.33 ± 4.37 a | 857.33 ± 7.42 a | 802 ± 53 a | 0.84 | 0.45 |
|  | mexI | 618 ± 43.19 a | 495.33 ± 65.34 b | 601.33 ± 32.36 ab | 567.33 ± 8.67 ab | 528 ± 24.44 ab | 0.32 | 1.29 |
|  | mexV | 229.33 ± 14.62 ab | 207.33 ± 3.33 abc | 244 ± 16.37 a | 198.67 ± 1.76 bc | 210.67 ± 18.27 abc | **0.05** | 2.76 |
|  | mexJ | 54.67 ± 7.69 a | 31.33 ± 5.81 b | 42 ± 3.46 ab | 52 ± 12.22 ab | 42 ± 2 ab | 0.32 | 1.30 |
|  | mexK | 694.67 ± 21.98 ab | 520.67 ± 42.98 d | 726 ± 30.55 a | 637.33 ± 8.51 bc | 590.67 ± 18.56 cd | **0.00** | 7.26 |
|  | mexP | 18.67 ± 3.71 bc | 22 ± 2 ab | 16.67 ± 1.76 bc | 19.33 ± 1.33 bc | 17.33 ± 1.76 bc | **0.02** | 3.64 |
|  | mexQ | 677.33 ± 55.92 a | 552.67 ± 32.99 b | 583.33 ± 13.92 ab | 638 ± 24.58 ab | 597.33 ± 32.54 ab | 0.18 | 1.76 |
|  | mexM | 38 ± 8 ab | 28.67 ± 8.74 b | 55.33 ± 9.33 a | 34 ± 3.06 b | 25.33 ± 4.06 b | 0.06 | 2.63 |
|  | mexN | 619.33 ± 9.33 a | 527.33 ± 39.94 b | 634 ± 38.57 a | 551.33 ± 7.42 ab | 582.67 ± 34.34 ab | 0.18 | 1.78 |
|  | mexW | 2097.33 ± 167.07 a | 1822 ± 65.43 b | 2118.67 ± 22.58 a | 1968 ± 13.32 ab | 1812 ± 114.02 b | 0.08 | 2.43 |
|  | mexY | 57.33 ± 7.69 a | 34.67 ± 2.4 a | 44.67 ± 13.48 a | 30.67 ± 8.67 a | 39.33 ± 8.82 a | 0.31 | 1.31 |
|  | oqxA | 376.67 ± 15.07 a | 359.33 ± 34.74 ab | 364.67 ± 20.08 ab | 330 ± 17.09 ab | 354.67 ± 18.41 ab | 0.42 | 1.09 |
|  | oqxB | 1098.67 ± 26.77 ab | 969.33 ± 64.76 b | 1139.33 ± 73.11 a | 1005.33 ± 56.36 ab | 970 ± 28.59 b | 0.17 | 1.80 |
|  | mexS | 121.33 ± 8.67 ab | 88 ± 6 b | 118.67 ± 10.73 ab | 133.33 ± 15.76 a | 112.67 ± 16.75 ab | 0.32 | 1.29 |
|  | drrA | 2005.33 ± 125.27 a | 1554 ± 108.3 b | 1878.67 ± 28.76 ab | 1814 ± 8.08 ab | 1799.33 ± 70.83 ab | 0.28 | 1.41 |
|  | mexT | 33.33 ± 4.81 a | 49.33 ± 3.71 a | 40 ± 10 a | 29.33 ± 4.67 a | 38 ± 4.62 a | 0.59 | 0.79 |
|  | mexF | 1821.33 ± 34.49 a | 1448 ± 70.21 bc | 1658 ± 43.19 ab | 1574 ± 72.04 bc | 1448 ± 121.08 bc | **0.02** | 3.91 |
|  | mdtC | 1586.67 ± 98.54 a | 1318.67 ± 98.42 b | 1555.33 ± 69.96 a | 1450.67 ± 17.75 ab | 1404.67 ± 41.46 ab | 0.07 | 2.58 |
|  | cmeB | 433.33 ± 22.52 a | 344.67 ± 51.44 ab | 396.67 ± 27.14 a | 348.67 ± 5.7 ab | 294.67 ± 32.67 b | 0.08 | 2.46 |
|  | macB | 6900.67 ± 325.7 a | 5651.33 ± 374.34 c | 6784 ± 28.1 ab | 6857.33 ± 94.38 ab | 6390.67 ± 283.7 abc | 0.06 | 2.70 |
|  | acrB | 144 ± 11.02 ab | 106 ± 9.24 c | 117.33 ± 8.67 abc | 127.33 ± 15.72 abc | 114 ± 2 bc | 0.16 | 1.84 |
| Other | cmlv | 72.67 ± 11.85 ab | 58.67 ± 7.69 b | 60.67 ± 2.67 b | 71.33 ± 13.38 ab | 95.33 ± 3.71 a | 0.12 | 2.11 |
|  | lnuF | 24 ± 6.11 a | 26 ± 2.31 a | 28.67 ± 11.22 a | 32 ± 5.77 a | 20 ± 3.46 a | 0.48 | 0.97 |
|  | Mycobacterium tuberculosis inhA mutations conferring resistance to isoniazid | 624 ± 55.51 a | 520.67 ± 8.11 a | 590.67 ± 42.96 a | 655.33 ± 27.72 a | 560.67 ± 38.86 a | 0.55 | 0.86 |
|  | clbA | 68 ± 10.58 bc | 72.67 ± 6.57 bc | 103.33 ± 15.38 a | 97.33 ± 5.33 ab | 72 ± 14.05 bc | 0.06 | 2.64 |
|  | clbB | 698.67 ± 52.84 a | 556.67 ± 61.73 b | 652 ± 16 ab | 666.67 ± 24.83 ab | 615.33 ± 10.73 ab | 0.22 | 1.61 |
|  | clbC | 170 ± 13.61 ab | 166 ± 14 abc | 132 ± 4.62 d | 177.33 ± 4.81 a | 140 ± 9.87 cd | **0.04** | 3.16 |
|  | cfrA | 48 ± 8.08 a | 31.33 ± 1.76 ab | 34.67 ± 9.68 ab | 38 ± 3.06 ab | 42.67 ± 11.68 ab | 0.35 | 1.23 |
|  | CIPa | 137.33 ± 13.38 a | 117.33 ± 5.21 a | 105.33 ± 15.68 a | 129.33 ± 1.76 a | 122 ± 7.57 a | 0.61 | 0.76 |
|  | mfpA | 24.67 ± 3.71 ab | 20.67 ± 2.67 b | 21.33 ± 2.91 b | 24 ± 6.11 ab | 33.33 ± 4.06 a | 0.31 | 1.32 |
|  | Escherichia coli mutant GlpT conferring resistance to fosfomycin | 89.33 ± 3.53 a | 49.33 ± 2.67 c | 50.67 ± 10.48 bc | 52.67 ± 5.93 bc | 52 ± 7.57 bc | **0.00** | 5.28 |
|  | desR | 217.33 ± 30.21 ab | 170.67 ± 13.38 b | 194.67 ± 2.91 ab | 251.33 ± 12.72 a | 229.33 ± 8.67 a | 0.15 | 1.91 |
|  | mycobacterium tuberculosis ethA mutant conferring resistance to ethionamide | 1070.67 ± 61.39 a | 884 ± 53.72 a | 1040 ± 11.02 a | 1028.67 ± 58.34 a | 970 ± 38.31 a | 0.38 | 1.16 |
|  | Mycobacterium tuberculosis ndh mutant conferring resistance to isoniazid | 2618 ± 112.26 a | 2164 ± 83.16 b | 2448 ± 66.4 ab | 2412.67 ± 55.69 ab | 2410 ± 143.25 ab | 0.36 | 1.21 |
|  | Mycobacterium tuberculosis kasA mutant conferring resistance to isoniazid | 3532 ± 206.07 a | 3120.67 ± 66.24 ab | 3342.67 ± 12.72 ab | 3244.67 ± 53.38 ab | 3090.67 ± 199.73 ab | 0.27 | 1.43 |
| Rifampin | rifampin phosphotransferase | 1462.67 ± 52.78 a | 1428 ± 52.62 a | 1440.67 ± 51.35 a | 1572.67 ± 34.65 a | 1450.67 ± 61.02 a | 0.79 | 0.52 |
|  | arr-1 | 44.67 ± 5.93 ab | 38 ± 4.16 ab | 28.67 ± 9.82 b | 36.67 ± 4.67 ab | 46.67 ± 5.81 ab | 0.39 | 1.14 |
|  | arr-4 | 16.67 ± 4.81 a | 11.33 ± 3.53 a | 8 ± 4.62 a | 9.33 ± 2.4 a | 6.67 ± 3.53 a | 0.49 | 0.95 |
|  | rgt1438 | 43.33 ± 14.53 a | 36.67 ± 2.4 a | 46.67 ± 4.37 a | 46.67 ± 11.79 a | 43.33 ± 8.19 a | 0.88 | 0.38 |
|  | iri | 789.33 ± 58.98 a | 622.67 ± 58.47 b | 718.67 ± 29.67 ab | 758 ± 16.37 ab | 705.33 ± 22.28 ab | 0.26 | 1.47 |
|  | Mycobacterium tuberculosis rpoB mutants conferring resistance to rifampicin | 3263.33 ± 156.8 a | 2800 ± 195.41 a | 3040 ± 34.7 a | 3038.67 ± 78.74 a | 2888.67 ± 132.21 a | 0.60 | 0.78 |
|  | Mycobacterium leprae rpoB mutants conferring resistance to rifampicin | 2098 ± 152.53 a | 1828.67 ± 67.06 a | 1967.33 ± 50.99 a | 1933.33 ± 59.58 a | 1958.67 ± 58.31 a | 0.40 | 1.11 |
|  | Staphylococcus aureus rpoB mutants conferring resistance to rifampicin | 1705.33 ± 56.77 a | 1637.33 ± 8.19 a | 1622.67 ± 113.81 a | 1606 ± 61.23 a | 1500 ± 116.37 a | 0.52 | 0.90 |
|  | Escherichia coli rpoB mutants conferring resistance to rifampicin | 4842.67 ± 137.7 a | 4022.67 ± 286.14 b | 4801.33 ± 275.4 a | 4364.67 ± 22.93 ab | 4286.67 ± 228.09 ab | 0.08 | 2.47 |
|  | Mycobacterium tuberculosis embB mutants conferring resistance to rifampicin | 40 ± 13.01 a | 27.33 ± 4.67 a | 38 ± 12.49 a | 60 ± 16.65 a | 33.33 ± 6.36 a | 0.48 | 0.96 |
| Sulfonamide | sul2 | 8 ± 5.03 a | 9.33 ± 2.4 a | 9.33 ± 2.91 a | 7.33 ± 0.67 a | 9.33 ± 2.4 a | 0.62 | 0.75 |
|  | Escherichia coli sulfonamide resistant mutant folP | 1320 ± 78.75 a | 1092 ± 74.14 b | 1196.67 ± 18.77 ab | 1264.67 ± 58.81 ab | 1205.33 ± 83.46 ab | 0.30 | 1.36 |
|  | Streptococcus pyogenes sulfonamide resistant folP mutants | 102.67 ± 13.97 a | 79.33 ± 4.06 a | 109.33 ± 5.7 a | 102.67 ± 9.82 a | 91.33 ± 8.97 a | 0.66 | 0.69 |
|  | Mycobacterium leprae dapsone resistant folP mutants | 546.67 ± 13.87 a | 490 ± 42 a | 534 ± 9.24 a | 572.67 ± 40.37 a | 590 ± 9.87 a | 0.48 | 0.97 |
|  | dfrA26 | 78.67 ± 6.77 a | 69.33 ± 9.4 ab | 74.67 ± 7.69 ab | 61.33 ± 1.33 bc | 58.67 ± 4.81 bc | **0.00** | 6.43 |
|  | dfrE | 610.67 ± 12.98 ab | 557.33 ± 40.5 b | 665.33 ± 24.34 a | 583.33 ± 7.42 b | 569.33 ± 12.24 b | **0.04** | 3.02 |
|  | dfrA3 | 184 ± 7.02 a | 169.33 ± 1.76 ab | 140 ± 25.01 b | 142.67 ± 10.09 ab | 134.67 ± 13.48 b | 0.20 | 1.67 |
| Tetracycline | tetO | 50 ± 5.03 a | 42 ± 12.22 a | 53.33 ± 12.72 a | 42.67 ± 2.91 a | 38.67 ± 8.97 a | 0.83 | 0.46 |
|  | tetQ | 27.33 ± 4.67 a | 22.67 ± 2.91 ab | 20 ± 4.16 ab | 25.33 ± 8.67 ab | 17.33 ± 1.33 ab | 0.37 | 1.19 |
|  | tetS | 83.33 ± 16.75 a | 57.33 ± 9.33 ab | 64 ± 3.06 a | 58.67 ± 10.97 ab | 61.33 ± 4.67 a | 0.07 | 2.52 |
|  | tetT | 772 ± 53.25 a | 750.67 ± 64.54 a | 766.67 ± 48.72 a | 822 ± 62.14 a | 756.67 ± 14.34 a | 0.92 | 0.31 |
|  | tetW | 72.67 ± 16.38 ab | 47.33 ± 3.71 b | 67.33 ± 9.61 ab | 60 ± 9.02 ab | 50 ± 6.43 b | 0.19 | 1.72 |
|  | tetB(P) | 717.33 ± 14.62 a | 628.67 ± 30.69 a | 718.67 ± 30.47 a | 698.67 ± 69.13 a | 642 ± 12.7 a | 0.37 | 1.18 |
|  | tet32 | 196.67 ± 17.94 a | 180 ± 14.47 a | 182.67 ± 13.53 a | 203.33 ± 13.97 a | 188.67 ± 8.19 a | 0.62 | 0.75 |
|  | tet36 | 36 ± 6.93 ab | 30 ± 10.07 b | 32.67 ± 2.91 ab | 47.33 ± 3.71 a | 44.67 ± 1.76 ab | 0.24 | 1.53 |
|  | tet44 | 78.67 ± 2.67 a | 44 ± 2 c | 64 ± 12.49 ab | 46.67 ± 2.4 bc | 62 ± 7.02 abc | **0.02** | 3.88 |
|  | otrA | 739.33 ± 26.19 a | 638.67 ± 54.46 a | 684.67 ± 29.24 a | 679.33 ± 21.46 a | 624.67 ± 19.68 a | 0.47 | 0.98 |
|  | rpsJ | 360 ± 21.01 a | 346.67 ± 33.65 a | 391.33 ± 18.05 a | 337.33 ± 4.67 a | 353.33 ± 8.51 a | 0.50 | 0.94 |
|  | tet34 | 121.33 ± 12.67 a | 95.33 ± 12.02 ab | 89.33 ± 7.86 b | 121.33 ± 10.97 a | 110 ± 6.11 ab | 0.29 | 1.37 |
|  | tetA | 23.33 ± 4.37 ab | 26.67 ± 3.71 ab | 14.67 ± 4.67 b | 30.67 ± 6.57 ab | 35.33 ± 9.61 a | 0.30 | 1.34 |
|  | tet30 | 15.33 ± 1.33 ab | 22 ± 6 a | 14 ± 1.15 ab | 9.33 ± 2.4 bc | 10.67 ± 4.67 bc | **0.03** | 3.25 |
|  | tet39 | 20 ± 8.33 a | 22.67 ± 2.4 a | 34 ± 4 a | 30 ± 9.87 a | 29.33 ± 7.86 a | 0.78 | 0.53 |
|  | tet41 | 107.33 ± 8.51 a | 62 ± 10.07 b | 100.67 ± 8.51 a | 102.67 ± 9.33 a | 102 ± 12.06 a | **0.04** | 3.17 |
|  | tet43 | 136.67 ± 14.53 ab | 117.33 ± 4.81 ab | 134.67 ± 11.79 ab | 148 ± 11.02 a | 125.33 ± 2.91 ab | 0.40 | 1.12 |
|  | tet35 | 60.67 ± 15.38 ab | 74 ± 6.11 ab | 57.33 ± 3.53 ab | 92 ± 8.08 a | 78.67 ± 1.76 ab | 0.29 | 1.37 |
|  | tetE | 6.67 ± 0.67 b | 8 ± 2 b | 8.67 ± 2.4 b | 12 ± 2.31 ab | 6.67 ± 1.33 b | **0.04** | 2.99 |
|  | tetA(P) | 236 ± 43.86 a | 210 ± 19.08 a | 214.67 ± 24.26 a | 232.67 ± 21.46 a | 198.67 ± 27.09 a | 0.79 | 0.52 |
|  | tetV | 399.33 ± 25.44 a | 362.67 ± 34.38 ab | 368 ± 4 ab | 400 ± 22.03 a | 368 ± 20.03 ab | 0.30 | 1.34 |

Note: A4PoW3, alfalfa-potato-winter wheat. BL, bare land; CWWM, corn-winter wheat-winter wheat-millet; PWWM, pea-winter wheat-winter wheat-millet; W, continuous winter wheat. Values within the same column followed by different letters indicate significant differences. Bold *P-*values indicate significant differences (*P*< 0.05; ANOVA, Tukey’s HSD test).

**Table S4** The abundance of antibiotic resistance genes at subtype level among distinct crop rotational regimes (n = 3). Values are mean ± standard deviation.

| **ARG type** | **A4PoW3** | **BL** | **CWWM** | **PWWM** | **W** | ***P*** | **F** |
| --- | --- | --- | --- | --- | --- | --- | --- |
| Aminocoumarin | 10279.33 ± 279.91 a | 8886 ± 316.44 c | 10227.33 ± 41.48 a | 10087.33 ± 113.27 ab | 9795.33 ± 392.79 abc | **0.04** | 2.99 |
| Aminoglycoside | 7061.33 ± 412.59 a | 5755.33 ± 289.99 b | 6566.67 ± 55.69 ab | 6674.67 ± 260.63 ab | 6249.33 ± 189.15 ab | 0.23 | 1.56 |
| Beta-lactam | 14887.33 ± 594.03 a | 12655.33 ± 570.16 c | 14467.33 ± 13.68 ab | 13982.67 ± 107.31 abc | 13743.33 ± 477.27 abc | 0.11 | 2.20 |
| Elfamycin | 6766.67 ± 335.28 a | 6099.33 ± 243.69 a | 6634.67 ± 90.34 a | 6414.67 ± 112.13 a | 6492.67 ± 325.05 a | 0.60 | 0.77 |
| Fluoroquinolone | 17430.67 ± 546.82 a | 14415.33 ± 610.26 c | 16180.67 ± 149.89 ab | 16245.33 ± 183.1 ab | 15326 ± 452.41 bc | **0.04** | 3.11 |
| Glycopeptide | 5658 ± 263.51 a | 4716.67 ± 264.2 a | 5254 ± 174.37 a | 5418 ± 185.11 a | 5091.33 ± 212.55 a | 0.38 | 1.16 |
| MLSB | 9263.33 ± 510.49 a | 7586.67 ± 360.12 b | 8824 ± 336.61 ab | 9216.67 ± 210.28 ab | 8338.67 ± 247.16 ab | 0.19 | 1.70 |
| Multidrug | 27394.67 ± 1164.42 a | 22632 ± 1320.11 c | 26514.67 ± 440.76 ab | 25480.67 ± 267.89 abc | 23894 ± 1030.76 bc | **0.05** | 2.86 |
| Rifampin | 14306 ± 356.02 a | 12452.67 ± 531.74 b | 13712 ± 272.54 ab | 13426 ± 243.33 ab | 12920 ± 511.01 ab | 0.19 | 1.73 |
| Sulfonamide | 2850.67 ± 112.62 a | 2466.67 ± 118.44 b | 2729.33 ± 46.67 ab | 2734.67 ± 97.77 ab | 2658.67 ± 76.81 ab | 0.24 | 1.54 |
| Tetracycline | 4260 ± 230.47 a | 3788.67 ± 113.54 a | 4081.33 ± 134.91 a | 4200 ± 172.06 a | 3904 ± 82.29 a | 0.47 | 0.98 |
| Other | 9394.67 ± 550.69 a | 7958.67 ± 261.71 b | 8804.67 ± 93.61 ab | 8881.33 ± 153.29 ab | 8453.33 ± 395.65 ab | 0.30 | 1.35 |
| Total | 120158 ± 4752.44 a | 101454.67 ± 4532.91 b | 115192 ± 419.01 ab | 113880.67 ± 1518.52 ab | 108413.33 ± 3905.89 ab | 0.11 | 2.13 |

Note: A4PoW3, alfalfa-potato-winter wheat. BL, bare land; CWWM, corn-winter wheat-winter wheat-millet; PWWM, pea-winter wheat-winter wheat-millet; W, continuous winter wheat. Values within the same column followed by different letters indicate significant differences. Bold *P-*values indicate significant differences (*P*< 0.05; ANOVA, Tukey’s HSD test).

**Table S5** The number of ARGs subtypes among distinct contrast group (n = 3).

| **ARG subtype** | **BL-W** | **BL-PWWM** | **BL-CWWM** | **BL-A4PoW3** |
| --- | --- | --- | --- | --- |
| Aminocoumarin | 2 | 1 | 2 | 1 |
| Aminoglycoside | 2 | 2 | 1 | 2 |
| Beta-lactam | 0 | 1 | 1 | 1 |
| Elfamycin | 1 | 0 | 0 | 1 |
| Fluoroquinolone | 1 | 2 | 1 | 5 |
| Glycopeptide | 1 | 3 | 2 | 6 |
| MLSB | 2 | 2 | 2 | 4 |
| Multidrug | 0 | 3 | 4 | 6 |
| Rifampin | 0 | 0 | 0 | 0 |
| Sulfonamide | 0 | 0 | 1 | 0 |
| Tetracycline | 0 | 1 | 1 | 2 |
| Other | 2 | 3 | 0 | 2 |

Note: A4PoW3, alfalfa-potato-winter wheat. BL, bare land; CWWM, corn-winter wheat-winter wheat-millet; PWWM, pea-winter wheat-winter wheat-millet; W, continuous winter wheat. BL-W, compare BL with W; BL-PWWM, compare BL with PWWM; BL-CWWM, compare BL with CWWM; BL- A4PoW3, compare BL with A4PoW3;

**Table S6** The representative resistance mechanisms among distinct crop rotational regimes (n = 3); values are mean ± standard deviation.

| Resistance mechanism | A4PoW3 | BL | CWWM | PWWM | W | Regimes *P* | Regimes F | Complexity *P* | Complexity F |
| --- | --- | --- | --- | --- | --- | --- | --- | --- | --- |
| Antibiotic target replacement | 2005.33 ± 42.85 a | 1811.33 ± 31.35 b | 1986.67 ± 68.53 a | 1835.33 ± 23.73 b | 1783.33 ± 8.19 b | **0.01** | 6.60 | **0.01** | 9.48 |
| Antibiotic deactivate | 3932 ± 187.89 a | 3472.67 ± 177.63 b | 3830 ± 39.72 ab | 4056 ± 75.8 a | 3816 ± 84.01 ab | 0.08 | 2.91 | 0.13 | 2.59 |
| Antibiotic target modification | 1122 ± 96.36 a | 944 ± 61.23 b | 1027.33 ± 39.91 ab | 1108.67 ± 28.88 ab | 992 ± 8.33 ab | 0.19 | 1.86 | 0.23 | 1.76 |
| Antibiotic target protection | 2224 ± 119.11 a | 1978 ± 102.07 a | 2170 ± 75.97 a | 2196 ± 130.5 a | 2044 ± 63.63 a | 0.41 | 1.09 | 0.26 | 1.63 |
| Cellular protection | 73548.67 ± 2720.46 a | 62306 ± 2555.01 c | 69912.67 ± 313.18 ab | 68932.67 ± 910.94 ab | 66586 ± 2590.14 bc | **0.03** | 4.02 | **0.04** | 4.28 |
| Efflux pump | 40399.33 ± 1973.42 a | 33331.33 ± 1742 c | 38851.33 ± 72.67 ab | 38313.33 ± 492.64 ab | 35597.33 ± 1358.24 bc | **0.03** | 4.39 | **0.04** | 4.44 |
| Other | 6321.33 ± 208.01 a | 5570 ± 240.21 b | 6218.67 ± 36.1 a | 6320 ± 111.22 a | 6048 ± 191.86 ab | 0.06 | 3.26 | 0.08 | 3.32 |

Note: A4PoW3, alfalfa-potato-winter wheat. BL, bare land; CWWM, corn-winter wheat-winter wheat-millet; PWWM, pea-winter wheat-winter wheat-millet; W, continuous winter wheat. Regimes, five crop rotational regimes; Complexity, four levels of crop rotation. Values within the same column followed by different letters indicate significant differences. Bold *P-values* indicate significant differences (*P*< 0.05; ANOVA, Tukey’s HSD test).

**Table S7** Soil properties in RDA significantly affect ARGs based on 999 permutation tests.

| Soil properties | R^2^ | *P* |
| --- | --- | --- |
| pH | 0.420 | 0.044 |
| NH^+^_4_-N | 0.164 | 0.350 |
| NO^-^_3_-N | 0.097 | 0.545 |
| SOC | 0.207 | 0.223 |
| PON | 0.223 | 0.196 |
| SWC | 0.281 | 0.144 |
| C/N | 0.466 | 0.028 |
| MBC | 0.426 | 0.022 |
| MBN | 0.418 | 0.038 |
| PCM/PNM | 0.118 | 0.477 |

Note: The fitting degree (R^2^) and the significance(P-values) of RDA model.

**Table S8** Correlations among the representative ARGs and the soil properties determined using the Mantel test.

|  | Glycopeptide | | Fluoroquinolone | | MLSB | | Multidrug | |
| --- | --- | --- | --- | --- | --- | --- | --- | --- |
| Soil properties | r | *P* | r | *P* | r | *P* | r | *P* |
| pH | 0.358 | **0.003** | 0.294 | **0.021** | 0.329 | **0.015** | 0.339 | **0.011** |
| NH_4_^+^-N | -0.152 | 0.793 | -0.170 | 0.822 | -0.089 | 0.654 | -0.224 | 0.946 |
| NO_3_^-^-N | -0.193 | 0.848 | -0.193 | 0.848 | -0.249 | 0.979 | -0.193 | 0.864 |
| SOC | 0.338 | **0.034** | 0.310 | **0.043** | 0.289 | **0.036** | 0.304 | **0.037** |
| PON | 0.058 | 0.318 | 0.043 | 0.347 | 0.078 | 0.279 | 0.055 | 0.371 |
| SWC | -0.029 | 0.504 | -0.095 | 0.656 | -0.075 | 0.611 | -0.006 | 0.459 |
| C/N | 0.513 | **0.001** | 0.554 | **0.001** | 0.457 | **0.003** | 0.576 | **0.001** |
| MBC | 0.057 | 0.269 | 0.023 | 0.298 | 0.193 | 0.178 | 0.006 | 0.341 |
| MBN | 0.316 | **0.011** | 0.319 | **0.006** | 0.291 | **0.011** | 0.358 | **0.006** |
| PCM/PNM | -0.171 | 0.820 | -0.172 | 0.832 | -0.201 | 0.941 | -0.086 | 0.642 |

Note: pH, soil pH; NH_4_^+^-N, ammonium nitrogen; NO_3_^-^-N,nitrate nitrogen; SOC, total organic carbon; PON, particulate organic nitrogen; SWC, soil water content; C/N, total carbon/nitrogen ratio; MBC, soil microbial biomass carbon; MBN, soil microbial biomass nitrogen; PCM/PNM, potential carbon mineralization/potential nitrogen mineralization ratio. The correlation coefficients (r) and significance (*P*-values) are provided. The *P*-values are derived from one-tailed tests based on 999 permutations. Bold *P-*values indicate significant differences (*P*< 0.05).

**Table S9** Correlations among the representative antibiotic resistance mechanisms and the soil properties determined using the Mantel test.

|  | Cellular protection | | Efflux pump | |
| --- | --- | --- | --- | --- |
| Soil properties | r | *P* | r | *P* |
| pH | 0.310 | **0.006** | 0.351 | **0.008** |
| NH_4_^+^-N | -0.170 | 0.848 | -0.196 | 0.895 |
| NO_3_^-^-N | -0.187 | 0.836 | -0.222 | 0.919 |
| SOC | 0.321 | **0.032** | 0.315 | **0.032** |
| PON | 0.031 | 0.375 | 0.069 | 0.337 |
| SWC | -0.096 | 0.677 | -0.031 | 0.483 |
| C/N | 0.560 | **0.001** | 0.573 | **0.001** |
| MBC | 0.041 | 0.290 | 0.058 | 0.258 |
| MBN | 0.318 | **0.005** | 0.360 | **0.004** |
| PCM/PNM | -0.173 | 0.819 | -0.127 | 0.727 |

Note: pH, soil pH; NH_4_^+^-N, ammonium nitrogen; NO_3_^-^-N, nitrate nitrogen; SOC, total organic carbon; PON, particulate organic nitrogen; SWC, soil water content; C/N, total carbon/nitrogen ratio; MBC, soil microbial biomass carbon; MBN, soil microbial biomass nitrogen; PCM/PNM, potential carbon mineralization/potential nitrogen mineralization ratio. The correlation coefficients (r) and significance (*P*-values) are provided. The *P*-values are derived from one-tailed tests based on 999 permutations. Bold *P-*values indicate significant differences (*P*< 0.05).

**Table S10** Node degree of soil properties and microbial biomass in the network.

| Soil properties | Node degree |
| --- | --- |
| pH | 49 |
| NH_4_^+^-N | 11 |
| NO_3_^-^-N | 10 |
| SOC | 53 |
| PON | 69 |
| SWC | 11 |
| C/N | 88 |
| MBC | 60 |
| MBN | 44 |
| PCM/PNM | 11 |

Note: pH, soil pH; NH_4_^+^-N, ammonium nitrogen; NO_3_^-^-N, nitrate nitrogen; SOC, total organic carbon; PON, particulate organic nitrogen; SWC, soil water content; C/N, total carbon/nitrogen ratio; MBC, soil microbial biomass carbon; MBN, soil microbial biomass nitrogen; PCM/PNM, potential carbon mineralization/potential nitrogen mineralization ratio.


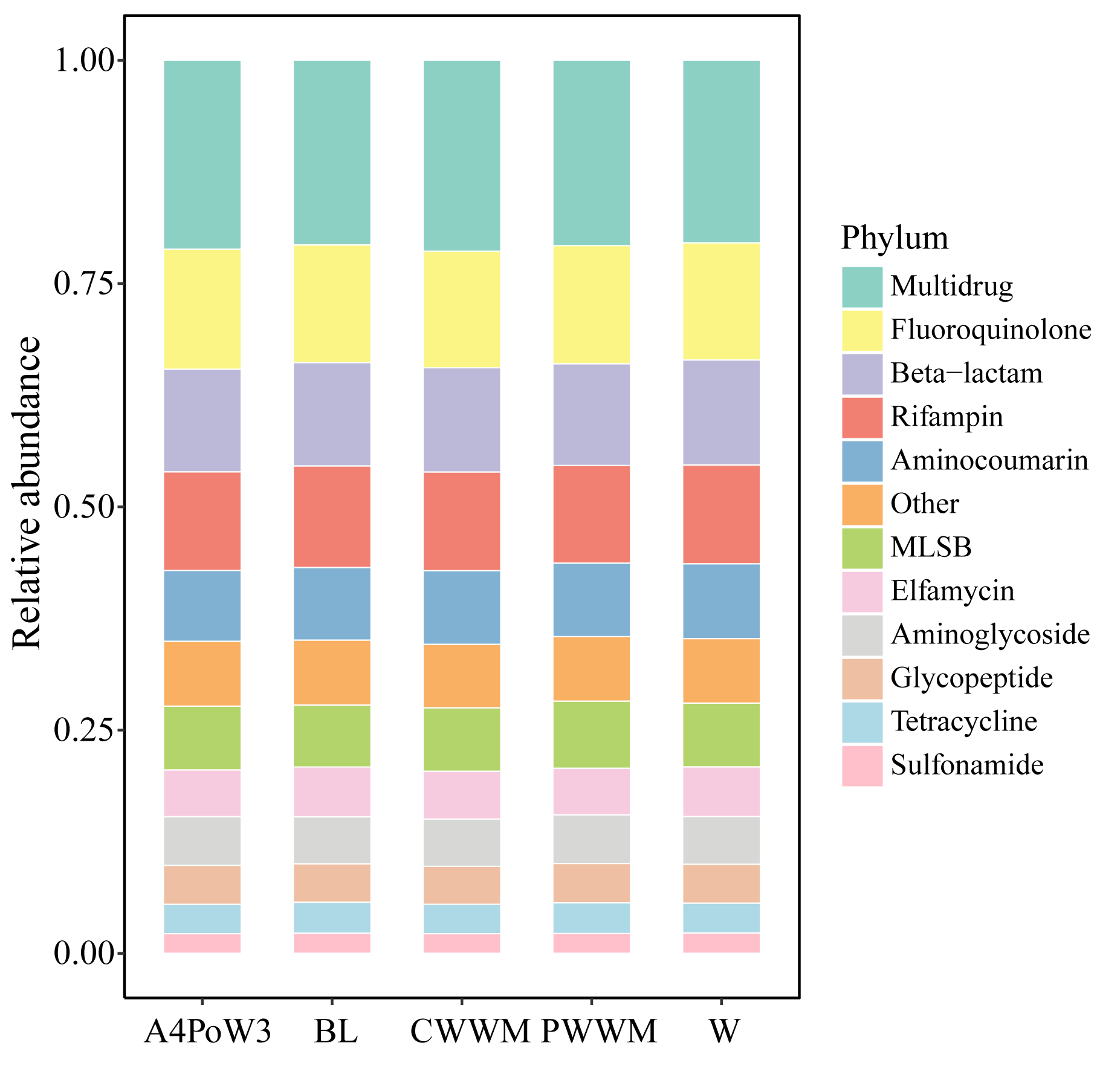


**Fig. S1** Relative abundance of ARGs types across distinct crop rotation regimes. The horizontal axis represents crop rotation regimes, the vertical axis represents relative abundance, and different colors indicate different types of ARGs. The legend is arranged in descending order based on relative abundance.


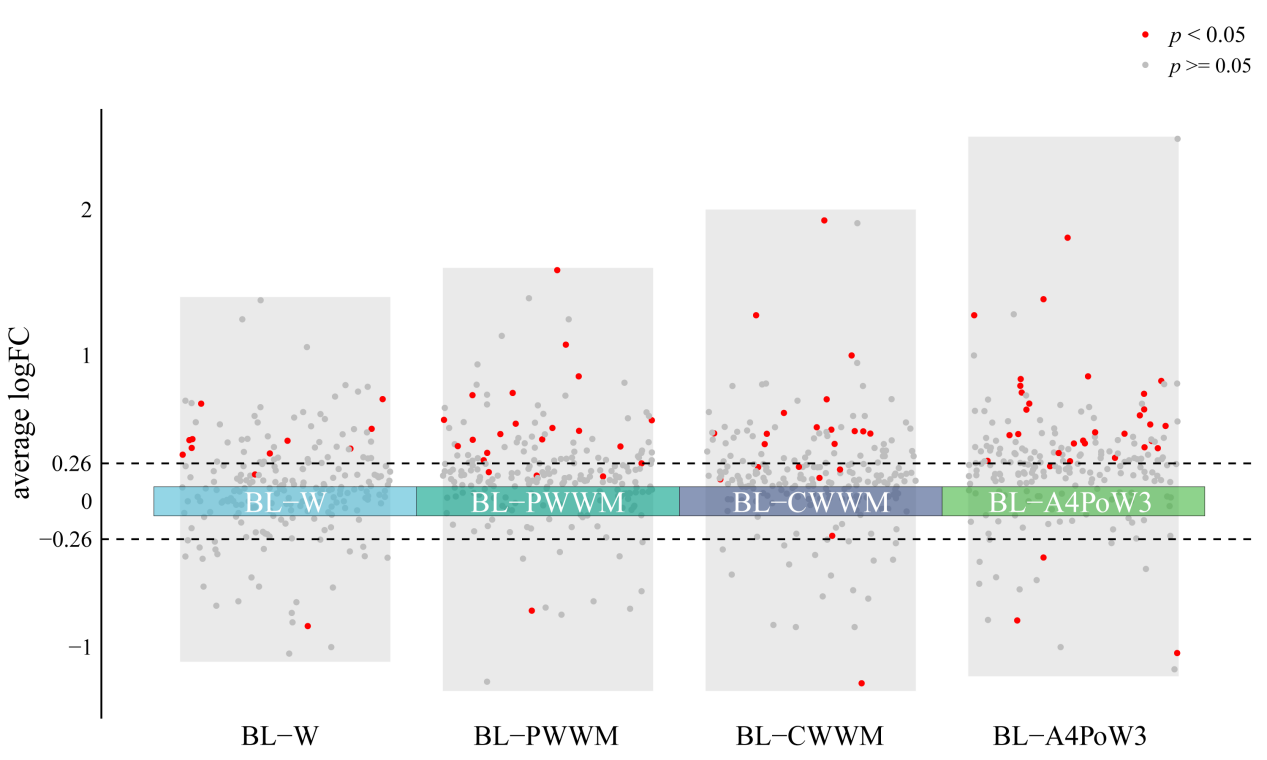


**Fig. S2** Volcano plot of differential resistance genes under different crop rotation systems, red scatters indicate *p* < 0.05, black scatters indicate *p* ≥ 0.05, and the four groups are the comparison groups between different crop rotation systems and control bare land. A4PoW3, alfalfa-potato-winter wheat. BL, bare land; CWWM, corn-winter wheat-winter wheat-millet; PWWM, pea-winter wheat-winter wheat-millet; W, continuous winter wheat.


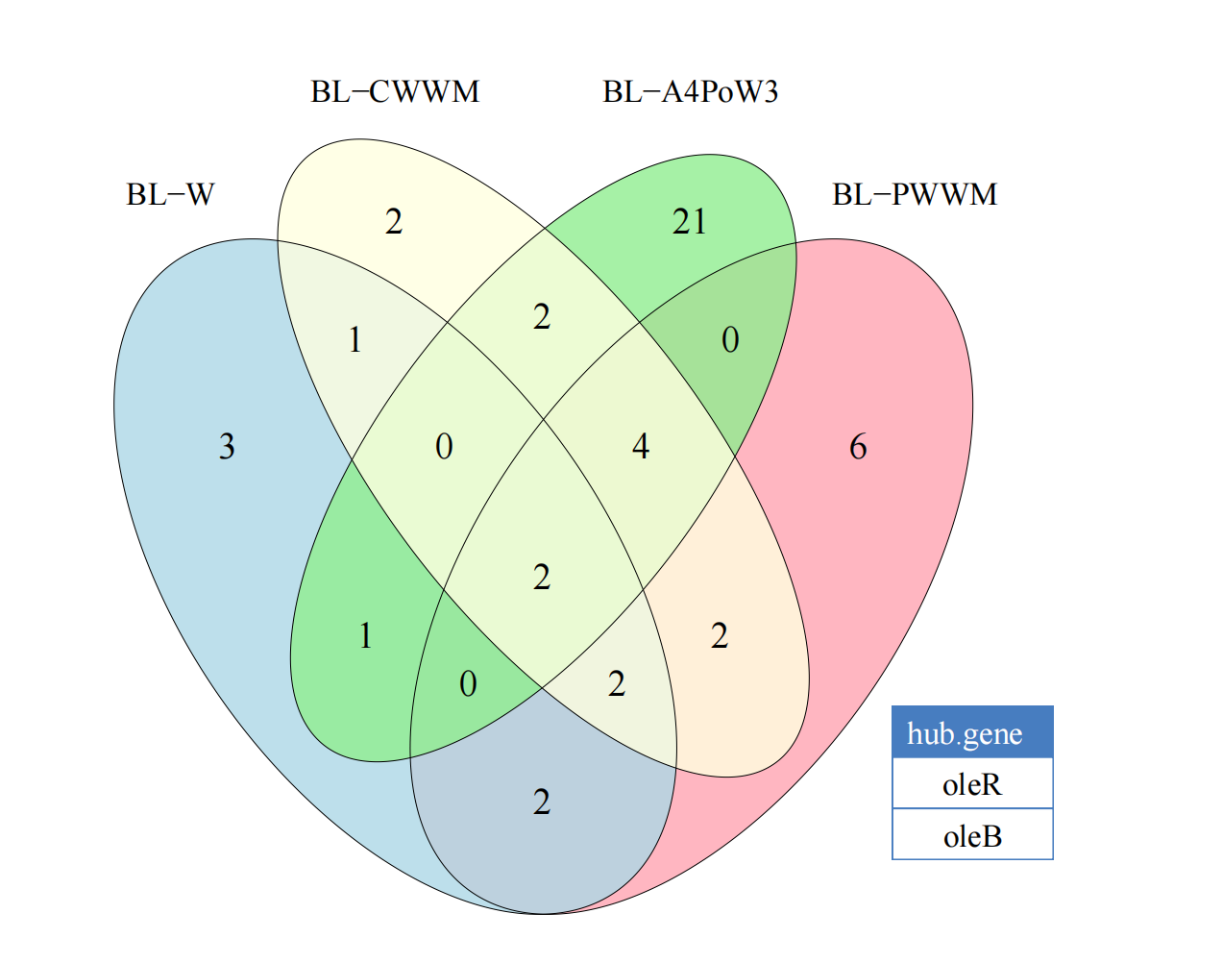


**Fig. S3** Veen plots of differential genes in different comparison groups. The four groups are the comparison groups between different crop rotation systems and control bare land.*oleR* and *oleB* are shared in all crop rotation regimes. A4PoW3, alfalfa-potato-winter wheat. BL, bare land; CWWM, corn-winter wheat-winter wheat-millet; PWWM, pea-winter wheat-winter wheat-millet; W, continuous winter wheat.


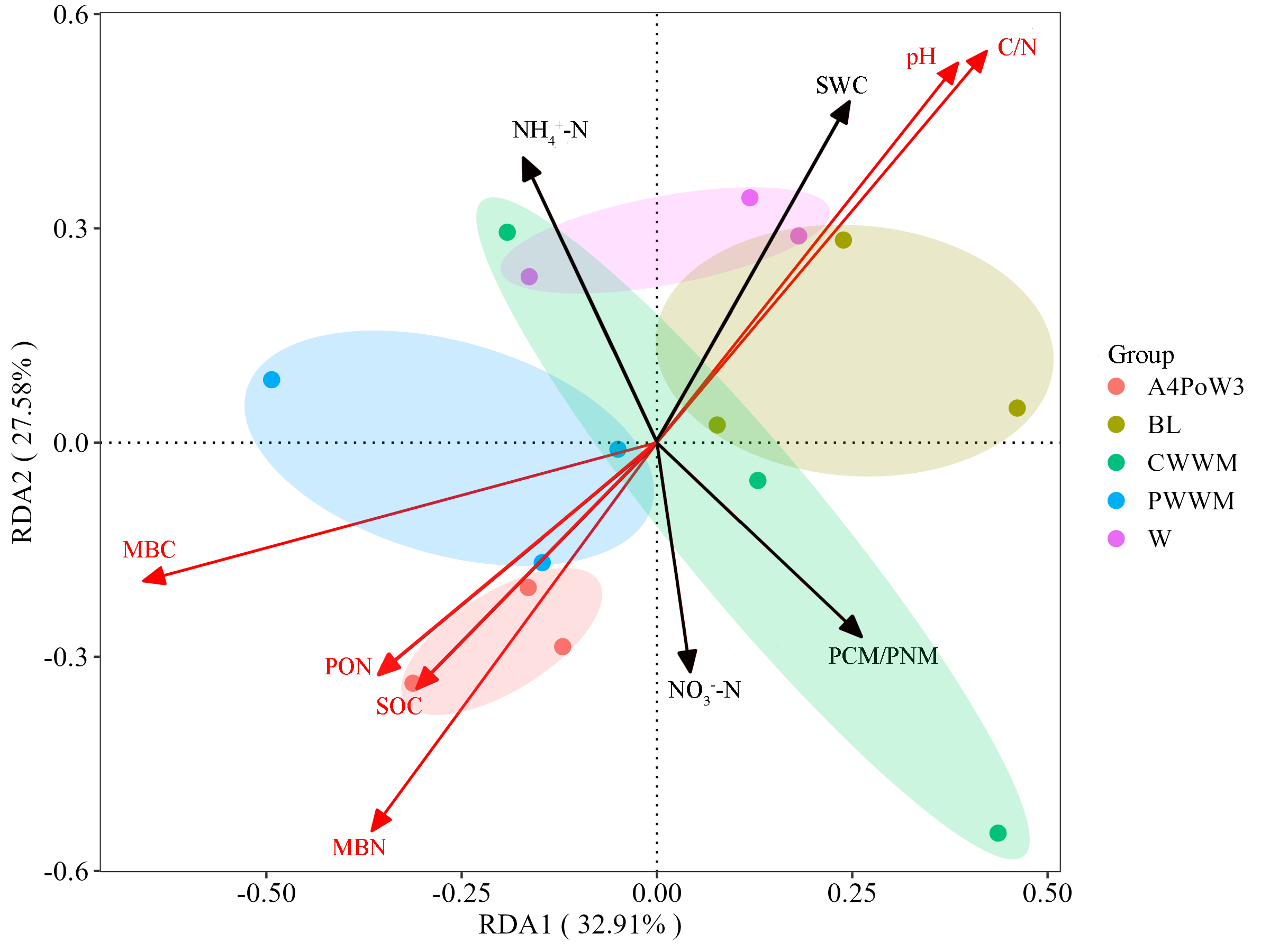
**Fig. S4** Redundancy analysis (RDA) between ARGs and soil properties under different crop rotation systems, with phase red arrows and font indicating important soil properties and black arrows and font indicating other soil properties. Each point represents a sample, and points of different colors belong to different groups. The angle between the arrows indicates the correlation between influencing factors. A4PoW3, alfalfa-potato-winter wheat. BL, bare land; CWWM, corn-winter wheat-winter wheat-millet; PWWM, pea-winter wheat-winter wheat-millet; W, continuous winter wheat. pH, soil pH; SWC, soil water content; NH_4_^+^-N, ammonium nitrogen; NO_3_^-^-N, nitrate nitrogen; and SOC, total organic carbon; MBC, soil microbial biomass carbon; MBN, soil microbial biomass nitrogen; C/N, total carbon to nitrogen ratio; PCM/PNM, potential carbon mineralization/potential nitrogen mineralization ratio; PON, particulate organic nitrogen.


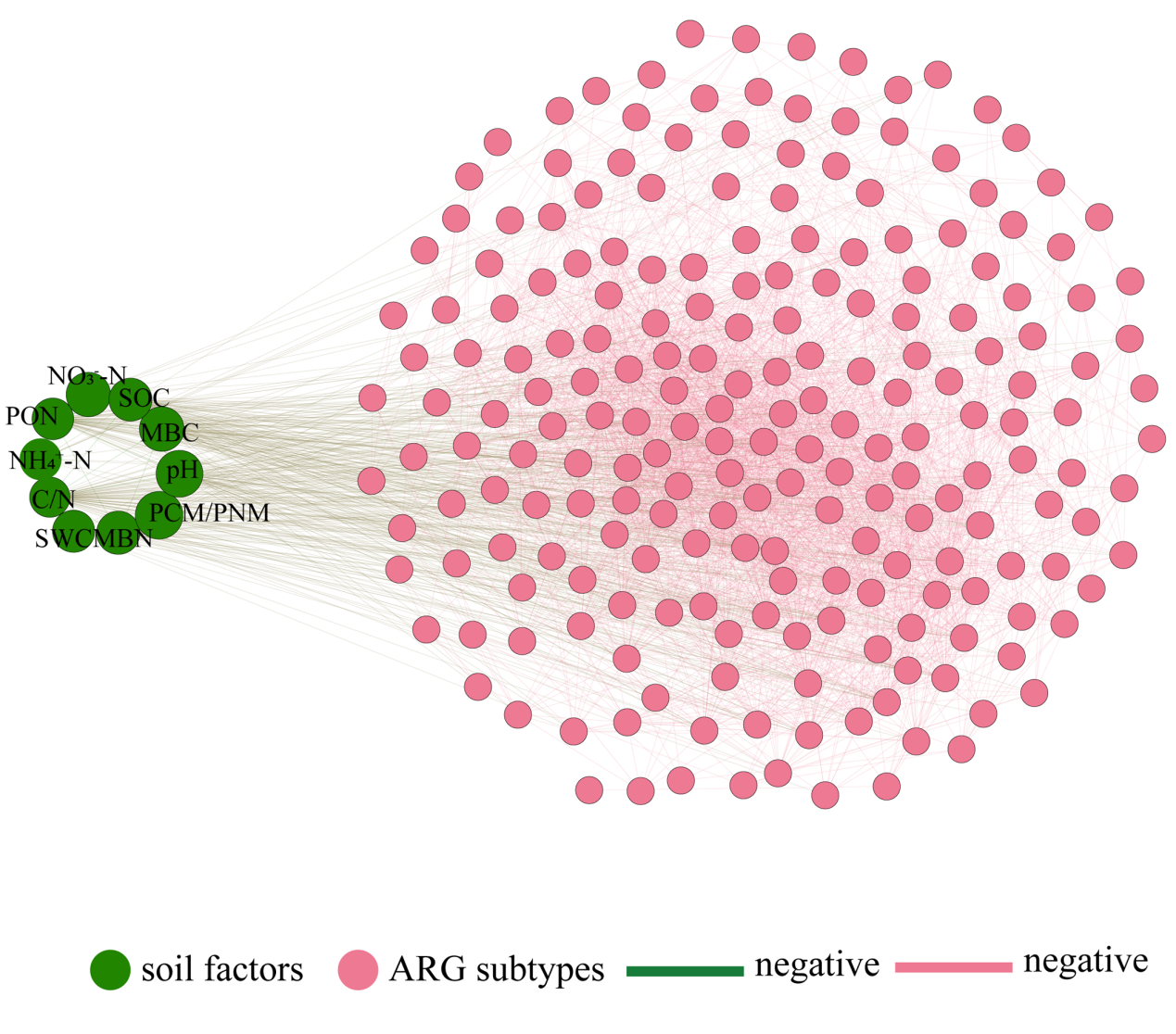


**Fig. S5** Networks between soil properties and microbial biomass and ARGs. Red nodes represent different ARG subtypes, and green nodes represent different soil properties and microbial biomass. Red lines indicate positive interactions and blue lines indicate negative interactions. pH, soil pH; SWC, soil water content; NH_4_^+^-N, ammonium nitrogen; NO_3_^-^-N, nitrate nitrogen; and SOC, total organic carbon; MBC, soil microbial biomass carbon; MBN, soil microbial biomass nitrogen; C/N, total carbon to nitrogen ratio; PCM/PNM, potential carbon mineralization/potential nitrogen mineralization ratio; PON, particulate organic nitrogen.
